# Xanthoepocin, a photolabile antibiotic of *Penicillium ochrochloron* CBS 123823 with high activity against multiresistant gram-positive bacteria

**DOI:** 10.1186/s12934-021-01718-9

**Published:** 2022-01-04

**Authors:** Pamela Vrabl, Bianka Siewert, Jacqueline Winkler, Harald Schöbel, Christoph W. Schinagl, Ludwig Knabl, Dorothea Orth-Höller, Johannes Fiala, Michael S. Meijer, Sylvestre Bonnet, Wolfgang Burgstaller

**Affiliations:** 1 Institute of Microbiology, University of Innsbruck, Technikerstraße 25d, 6020 Innsbruck, Austria; 2 Institute of Pharmacy/Pharmacognosy, Center for Molecular Biosciences Innsbruck (CMBI), Center for Chemistry and Biomedicine, University of Innsbruck, Innrain 80-82, 6020 Innsbruck, Austria; 3 MCI - The Entrepreneurial University, Maximilianstraße 2, 6020 Innsbruck, Austria; 4 Division of Hygiene and Medical Microbiology, Medical University of Innsbruck, Schöpfstraße 41, 6020 Innsbruck, Austria; 5 Present Address: Tyrolpath Obrist Brunhuber GmbH, Hauptplatz 4, 6511 Zams, Austria; 6 Present Address: MB-Lab, Clinical Microbiology Laboratory, Franz Fischer Str. 7b, 6020 Innsbruck, Austria; 7 Leiden Institute of Chemistry, Leiden University, Einsteinweg 55, 2333CC Leiden, The Netherlands; 8 Present Address: Department of Chemical Engineering, Delft University of Technology, Van der Maasweg 9, 2629HZ Delft, Netherlands

**Keywords:** Light, Filamentous fungi, Reexamination of a known secondary metabolite, Light-dependent produced secondary metabolite, MRSA, LVRE, Photoantimicrobial test, PDT, ROS

## Abstract

**Background:**

With the steady increase of antibiotic resistance, several strategies have been proposed in the scientific community to overcome the crisis. One of many successful strategies is the re-evaluation of known compounds, which have been early discarded out of the pipeline, with state-of-the-art know-how. Xanthoepocin, a polyketide widespread among the genus Penicillium with an interesting bioactivity spectrum against gram-positive bacteria, is such a discarded antibiotic. The purpose of this work was to (i) isolate larger quantities of this metabolite and chemically re-evaluate it with modern technology, (ii) to explore which factors lead to xanthoepocin biosynthesis in *P. ochrochloron*, and (iii) to test if it is beside its known activity against methicillin-resistant *Staphylococcus aureus* (MRSA), also active against linezolid and vancomycin-resistant *Enterococcus faecium* (LVRE)—a very problematic resistant bacterium which is currently on the rise.

**Results:**

In this work, we developed several new protocols to isolate, extract, and quantify xanthoepocin out of bioreactor batch and petri dish-grown mycelium of *P. ochrochloron*. The (photo)chemical re-evaluation with state-of-the-art techniques revealed that xanthoepocin is a photolabile molecule, which produces singlet oxygen under blue light irradiation. The intracellular xanthoepocin content, which was highest under ammonium-limited conditions, varied considerably with the applied irradiation conditions in petri dish and bioreactor batch cultures. Using light-protecting measures, we achieved MIC values against gram-positive bacteria, including methicillin-resistant *Staphylococcus aureus* (MRSA), which were up to 5 times lower than previously published. In addition, xanthoepocin was highly active against a clinical isolate of linezolid and vancomycin-resistant *Enterococcus faecium* (LVRE).

**Conclusions:**

This interdisciplinary work underlines that the re-evaluation of known compounds with state-of-the-art techniques is an important strategy in the combat against multiresistant bacteria and that light is a crucial factor on many levels that needs to receive more attention. With appropriate light protecting measures in the susceptibility tests, xanthoepocin proved to be a powerful antibiotic against MRSA and LVRE. Exploring the light response of other polyketides may be pivotal for re-introducing previously discarded metabolites into the antibiotic pipeline and to identify photosensitizers which might be used for (antimicrobial) photodynamic therapies.

**Supplementary Information:**

The online version contains supplementary material available at 10.1186/s12934-021-01718-9.

## Background

With multi-drug resistant bacteria on the rise and, at the same time, a dwindling antibiotic pipeline, the necessity for novel antibiotics is more extensive than ever before. Multiple strategies have been suggested to overcome the antibiotic crisis. Amongst others, these include raising global public awareness, preventive hygienic measures, a more targeted use of antibiotics in agriculture and medicine, and the search for novel therapies and bioactive compounds [1]. One other promising strategy is reexamining already known microorganism-derived antibacterial natural products, which were formerly excluded in the first stages from the screening process because they did not meet criteria like broad spectrum activity, efficacy, stability, or lack of cytotoxicity [2–4]. Consequently, most of these compounds were never explored in more depth again [4] and ended up on the “dusty shelves” of universities or pharmaceutical companies [2].

A systematic reexploration of this unexploited reservoir of antibiotics is often difficult. For instance, most of these metabolites are commercially unavailable and have to be isolated anew for more extensive test series. Nevertheless, the re-investigation of formerly discarded metabolites such as daptomycin or fidaxomicin eventually led to a successful therapeutic implementation of these antibiotics [2]. Other recent findings, such as the reexamination of γ-actinorhodin, underline this approach’s potential further [3].

**Fig. 1:**
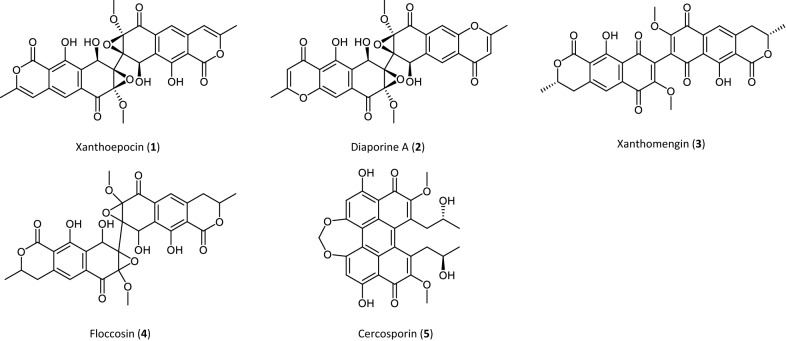
Structural formula of xanthoepocin and its related structures. Stereochemical information is depicted where known

The polyketide xanthoepocin (Fig. 1, **1**), a member of the xanthomegnin-family, is a typical example of such an early discarded metabolite with interesting bioactive properties. The yellow pigment is widespread in Penicillia [5–10] and was first isolated by Igarashi and co-workers in 2000 [11]. Due to its interesting bioactivity spectrum against pathogenic yeasts and methicillin-resistant *Staphylococcus aureus* (MRSA) combined with weak cytotoxicity against histiocytic lymphoma (U937) [11], xanthoepocin even entered the preclinical test phase in the same year [12]. Nevertheless, no further information regarding this matter is available in literature.

**Fig. 2:**
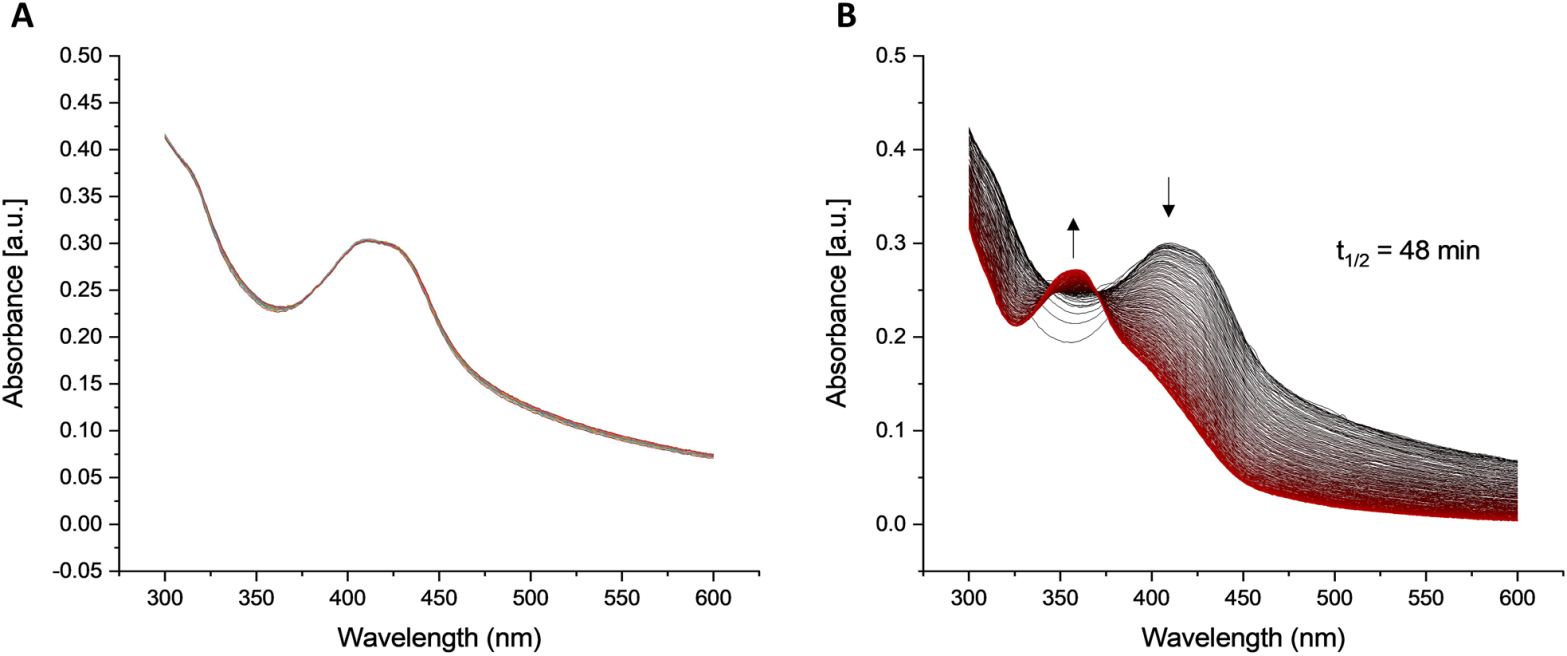
Evolution of the UV–Vis absorption spectrum of xanthoepocin in water. **A** The sample was kept in the dark. **B** The sample was irradiated with blue light (413 nm, 5 J cm^−2^) perpendicularly to the beam of the UV–Vis spectrophotometer

Xanthoepocin (**1**) has two known analogs, namely floccosin (Fig. 1, **4**) and diaporine A (Fig. 1, **2**), which share with it the typical semiquinone tetragonal structures with epoxide rings. Floccosin and diaporine A are also bioactive metabolites: floccosin, isolated from the pathogenic fungus *Epidermophyton floccosum* in the 1960s [13, 14], was reported to have an uncoupling effect on isolated mitochondria [15]. Despite its structural similarity to xanthomegnin (Fig. 1,** 3**), it appears to be no genotoxic carcinogen [16, 17]. Diaporine A was only recently discovered in culture broths of the endophytic fungus *Diaporthe sp. 3lp-10* [18, 19] and became a focus of scientific interest because of its anticancer activity [18, 20]. A recent survey on the meta-level highlighted the enormous pharmaceutical potential of di-epoxic compounds such as xanthoepocin as these metabolites show a wide bioactivity spectrum [21].

After the initial study of Igarashi et al. [11] using *P. simplicissimum*, xanthoepocin was identified as metabolite of several other Penicillium species (e.g. [5–10]) and a biosynthetic route was suggested [22, 23]. Just recently, the absolute configuration was resolved [24]. So far, however, trigger factors leading to its production are completely unknown, and also the biological function of this widespread metabolite still remains in the dark. While Igarashi and colleagues [11] reported the effectiveness of xanthoepocin against MRSA, this was neither confirmed again, nor was it tested against other multiresistant gram-positive bacteria.

The present work's initial purpose was to explore the bioactivity and physiological requirements leading to xanthoepocin biosynthesis in *Penicillium ochrochloron*. In the course of the isolation process and the development of a targeted HPLC-analysis, we unexpectedly found that xanthoepocin is photolabile. This observation raised the question, whether or not its biosynthesis is also light-dependent. In the present study we demonstrate that ammonium limitation and red light or darkness, respectively, foster high intracellular xanthoepocin concentrations in this organism. In addition to a more in-depth (photo)chemical characterization of xanthoepocin, we found that the published MIC concentrations underestimate this metabolite's effectiveness against gram-positive bacteria including MRSA by factors up to 5—because its light-sensitivity was not taken into consideration up to now. We furthermore demonstrate that xanthoepocin is also effective against clinical isolates of linezolid and vancomycin-resistant *Enterococcus faecium*.

## Results

### (Photo)chemical characterization of xanthoepocin

#### Isolation of xanthoepocin

Starting with dried mycelium (cultivated without controlled irradiation conditions), an optimized extraction process of xanthoepocin was achieved. In brief, the ground mycelium was defatted with petroleum ether and afterward extracted with acetone. The obtained ochreous and viscous extract (m_acetone-extract_ = 461.1 mg, η = 0.71% dry weight) contained xanthoepocin and aliphatic, probably oily, components as identified by H-NMR (data not shown). The extract was submitted to flash chromatography yielding an enriched fraction of xanthoepocin (m = 142.6 mg, η = 0.22% dry weight, purity 92% (HPLC)). Proton NMR spectroscopy revealed unsaturated fatty acids as impurities (data not shown). Ultrapure xanthoepocin (purity > 99% HPLC) was obtained via preparative HPLC as light-yellow solid (m = 69.16 mg, η = 0.11% dry weight). Starting from the extract, the procedure yielded η = 15% xanthoepocin, while previously reported yields equaled η = 1.5%, which is 10 times lower [11].

#### Xanthoepocin quantification method

Based on the ICH guidelines [25], a quantitative analysis on a HPLC–DAD system was established. After an exhaustive extraction of the finely ground and sieved mycelium, the xanthoepocin concentration was determined by HPLC analysis (t_ret_ = 12 min) via an external calibration (Additional file 1: Fig. S1). The calibration curve was linear between c = 0.1 and 0.5 mg mL^−1^ and characterized by a correlation coefficient (R^2^) of 0.9999 (Additional file 1: Fig. S1). The limit-of-detection (LOD) and the limit-of-quantification (LOQ) were calculated based on the standard deviation of the calibration curve (SD_Slope_, ICH guideline) to be 0.005 µg mL^−1^ and 0.014 µg mL^−1^, respectively. Via a classic visual determination method, different values were found: While the LOD was determined to be 0.29 µg mL^−1^, the LOQ was 0.97 µg mL^−1^. During the entire process, exposition to light was omitted. The accuracy of the instrument was determined to be > 99%. The repeatability of the extraction was determined with an interday and intraday precision of 6% and 5% (RSD), respectively.

#### Chemical characterization

Xanthoepocin was characterized by the means of NMR (Additional file 1: Fig. S2), infrared (Additional file 1: Fig. S3), and UV–Vis spectroscopy (Additional file 1: Fig. S4), as well as mass spectrometry (MS). Furthermore, the melting point and retention factor were determined. The results can be found in the Additional file 1: Material in Chapter 1. Via comparison with the available data from the literature (NMR and MS [11, 24]) and interpretation of the newly recorded data, we could confirm the isolation of xanthoepocin from *P. ochrochloron*.

Furthermore, the aggregation behavior and the stability of xanthoepocin were studied. The latter revealed that light had a degrading effect on xantheopocin (Fig. 2), it was therefore studied in more detail (see “Effect of light on Xanthoepocin” Section). In addition, in Müller-Hinton broth medium, the formation of aggregates was observed by dynamic light scattering (Additional file 1: Fig. S5). As this medium is a multi-component mixture containing casein, we decided to study the aggregation behavior of xanthoepocin in phosphate-buffered saline (PBS). The critical aggregation concentration (CAC) was calculated to be 10 µM via plotting the intensity of light scattering (in kilo counts per second (kcps)) *vs* the concentration of xanthoepocin (Additional file 1: Fig. S6).

#### Effect of light on xanthoepocin

##### Decomposition under sunlight and controlled light irradiation

A HPLC–DAD analysis of a xanthoepocin solution (0.47 mg mL^−1^ in DMSO, 0.77 mM) was performed before and after sunlight exposure (Additional file 1: Fig. S7) and revealed the kinetic formation of several irradiation products. For a more controlled process, a solution of xanthoepocin (12.5 µg mL^−1^) was irradiated in DMSO, and an aliquot withdrawn every minute. The formation of the first photoproduct could be observed after two minutes of irradiation (Additional file 1: Fig. S9). Within 20 min, over ten different photoproducts were found. In a next step, the light-induced total degeneration of xanthoepocin was spectroscopically studied (λ = 413 nm, 5 J cm^−2^, 90 min, Water/3% DMSO, 33 µM xanthoepocin) and a half-life time (t_1/2_) of 48 min was determined (Fig. 2 and Additional file 1: Fig. S8). As control served the same solution (xanthoepocin, 33 µM, H_2_O) under dark conditions. As depicted in Fig. 2, xanthoepocin was stable in the dark, while under blue light irradiation, it degenerated.

##### Singlet oxygen test

To test whether xanthoepocin and/or its decomposition products can produce reactive oxygen species, and more specifically, singlet oxygen (^1^O_2_), a 9,10-dimethylanthracene (DMA) assay [26] was performed. Singlet oxygen oxidizes DMA via the formation of an endoperoxide yielding 9,10-endoperoxianthracene and thus interrupting the π-electron conjugated system within the DMA molecule. In consequence, the typical near-UV absorbance pattern of DMA (λ_max_ = 377 nm) is lost. The irradiation (λ = 468 nm, 20 J cm^−2^, 15 min) of xanthoepocin led indeed to the reduction of the DMA absorption, indicating the production of ^1^O_2_. Ascorbic acid, a reductant of reactive oxygen species (ROS), was able to quench the DMA oxidation and thus proved the involvement of singlet oxygen. To quantify the singlet oxygen production by xanthoepocin, we recorded the emission of singlet oxygen at λ_em_ = 1270 nm in an aerated solution of xanthoepocin. The photochemical singlet oxygen production quantum yield (Φ_Δ_) was determined to be 0.078 (measured relative to [Ru(bpy)_3_]^2+^ (Φ_Δ_ = 0.73)) in deuterated methanol. Time-course studies revealed that the ability of a xanthoepocin solution to produce singlet oxygen under irradiative conditions declined with time (Additional file 1: Fig. S11). A loss of twentyfive percent was detected within the first 15 min of irradiation. Furthermore, the fluorescent properties of xanthoepocin were studied in the same solvent (d4-MeOH, Additional file 1: Fig. S10). Xanthoepocin emitted at λ_max_ = 516 nm with a fluorescence quantum yield of Φ_em_ = 0.0099.

### Solid state cultures—petri dish experiments

The light sensitivity of xanthoepocin raised the question of whether or not its biosynthesis is also light-dependent, as it is known that the polyketide pathway is susceptible to light [27]. To receive a first idea, we performed an explorative screening with petri dish cultures using a broad irradiation spectrum, i.e., central wavelengths from 394 ± 10 nm to 631 nm ± 17 nm (Additional file 1: Table S2) and cultivation in darkness as control. Since it is well-known that the nutritional state is decisive for light responses and secondary metabolite production [27, 28], the experiment was performed with three different minimal media, leading to glucose, ammonium, or phosphate limitation.

**Fig. 3:**
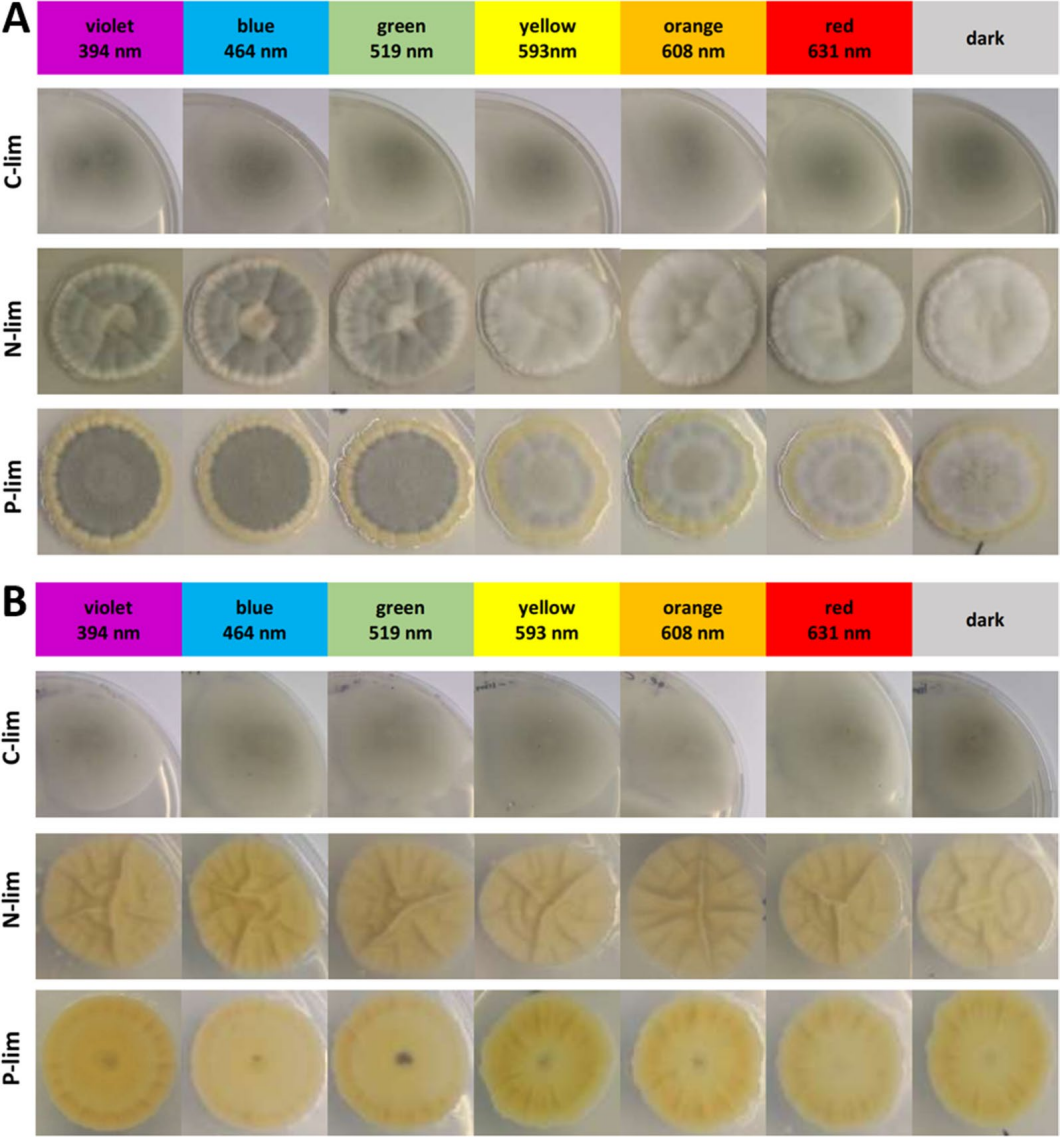
Nutrient-dependent light response of *Pencillium ochrochloron* CBS123823 grown for 7 days at 25 °C on glucose-, ammonium-, or phosphate-limited agar medium under either constant illumination (central wavelengths are given) or in darkness. **A** Front side and **B** reverse side of a typical colony per illumination condition are shown

*P. ochrochloron* showed a distinctive, nutrient-dependent light response in terms of sporulation, pigmentation, and colony diameter (Fig. 3, Additional file 1: Fig. S14): glucose-limited grown cultures, which showed the largest colony diameters, were unpigmented and sporulated under all tested conditions. In contrast, colonies of phosphate or ammonium-limited cultures were considerably smaller, and their pigmentation and sporulation pattern changed substantially with the applied wavelength, e.g., the fungus only sporulated in cultures subjected to wavelengths of 519 nm and below.

**Fig. 4:**
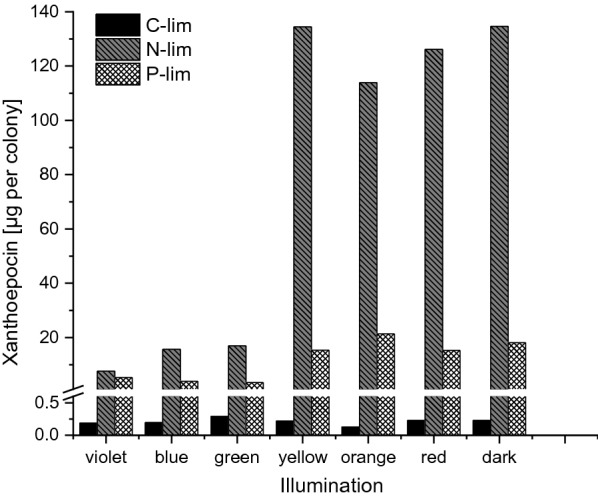
Xanthoepocin content of *Pencillium ochrochloron* CBS123823 colonies grown for 7 days at 25 °C on glucose-, ammonium-, or phosphate-limited agar medium under either constant illumination or in darkness in an explorative experiment

The xanthoepocin concentration was clearly dependent on the irradiation and nutritional conditions (Fig. 4): Glucose-limited grown colonies contained almost no xanthoepocin regardless of the illumination conditions. In contrast, phosphate and ammonium limited cultures showed an increased xanthoepocin content when the cultures were either illuminated with yellow, orange, or red light or grown in complete darkness. The highest xanthoepocin content was present in ammonium-limited cultures, although phosphate-limited cultures appeared more yellow. A HPLC analysis indicated that this was due to the quantity of blue-light absorbing metabolites other than xanthoepocin, which were higher in phosphate-limited cultures (n = 14) than in ammonium-limited cultures (n = 6) (Additional file 1: Fig. S15). Based on these results, ammonium-limitation was chosen as condition for the subsequent bioreactor batch cultivations.

### Submerged cultures—bioreactor batch experiments

**Fig. 5:**
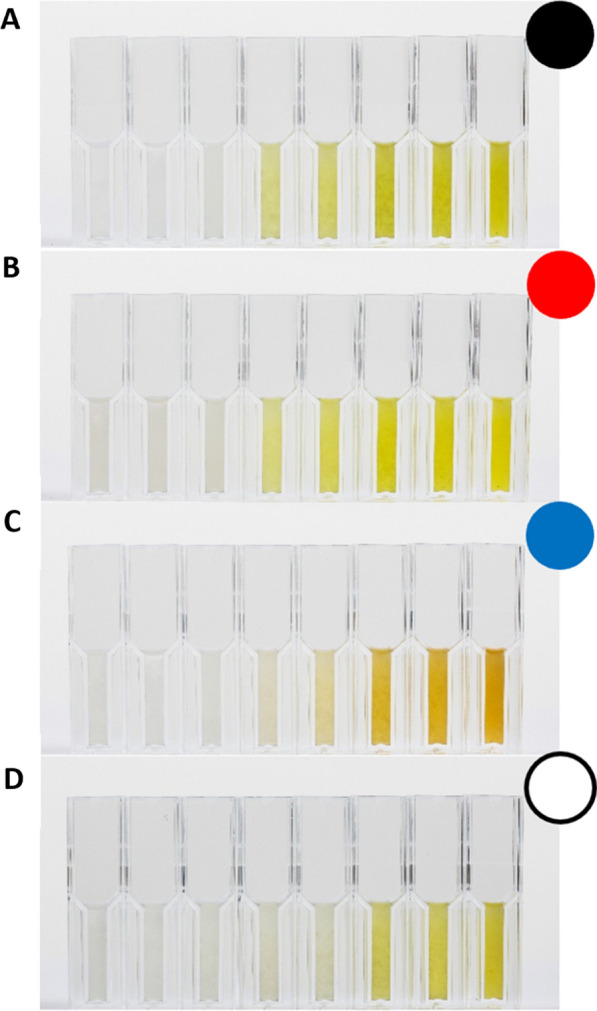
Typical progress of culture broth pigmentation of ammonium limited grown *Pencillium ochrochoron* CBS123823 bioreactor batch cultures in dependence on the illumination conditions. Cuvettes show the single sampling time points in ascending chronology, i.e. cuvette *left* sampling time point t = 0 h, cuvette *right* sampling time point t = 90 h. **A** darkness, **B** red light (λ_peak_ = 660 nm), **C** blue light (λ_peak_ = 451 nm), **D** ambient (white) light (4100 K)

**Fig. 6:**
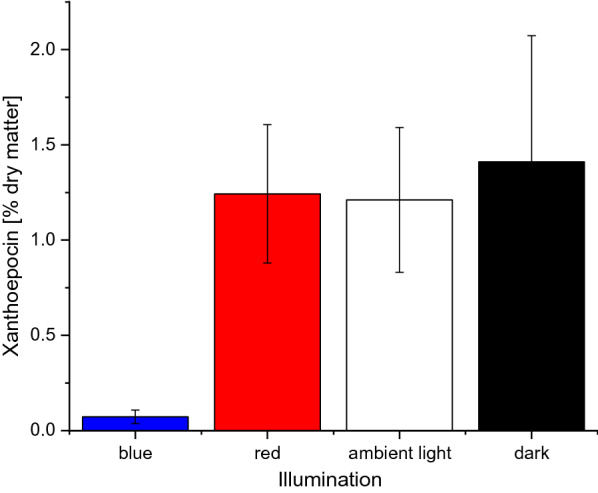
Xanthoepocin content of ammonium limited grown bioreactor batch cultures of *Pencillium ochrochloron* CBS123823 after approximately 90 h constant illumination (details see Additional file 1: Table S3) or darkness. Data represent the means of at least two independent bioreactor batch cultivations whereby each sampling point consisted of triplicate samples. Error bars indicate the standard deviation

Since the petri dish experiments indicated that ammonium limitation in combination with incubation at higher wavelengths or in darkness increased the intracellular xanthoepocin concentration in *P. ochrochloron*, these conditions were used for the subsequent bioreactor batch experiments. Independent of the illumination conditions, *P. ochrochloron* exhibited the previously described growth curve for this medium, which consisted of a short exponential growth phase followed by an extended secondary growth phase (e.g., [29]; Additional file 1: Figs. S17–S20). The typical yellowish, slightly greenish coloration attributed to xanthoepocin [11] was visible after the onset of ammonium limitation in cultures grown in the dark, red light, or ambient light conditions (Fig. 5 A, B, D). In contrast, cultures subjected to blue light showed a distinct orange pigmentation. This change in pigmentation was also reflected in the xanthoepocin content (Fig. 6): while the dry weight of cultures grown in the dark, red light, or ambient light conditions contained approximately 1.2% (w/w%) xanthoepocin, blue light exposure reduced the xanthoepocin content below 0.1% (w/w%) of the dry weight. Thus, the bioreactor batch experiments underlined the observations of the screening experiment with petri dish cultures: Conditions which appeared to increase the xanthoepocin content in *P. ochrochloron* are ammonium limitation and wavelengths above 595 nm or darkness. Exposure to blue light drastically reduced the intracellular xanthoepocin concentration.

**Fig. 7:**
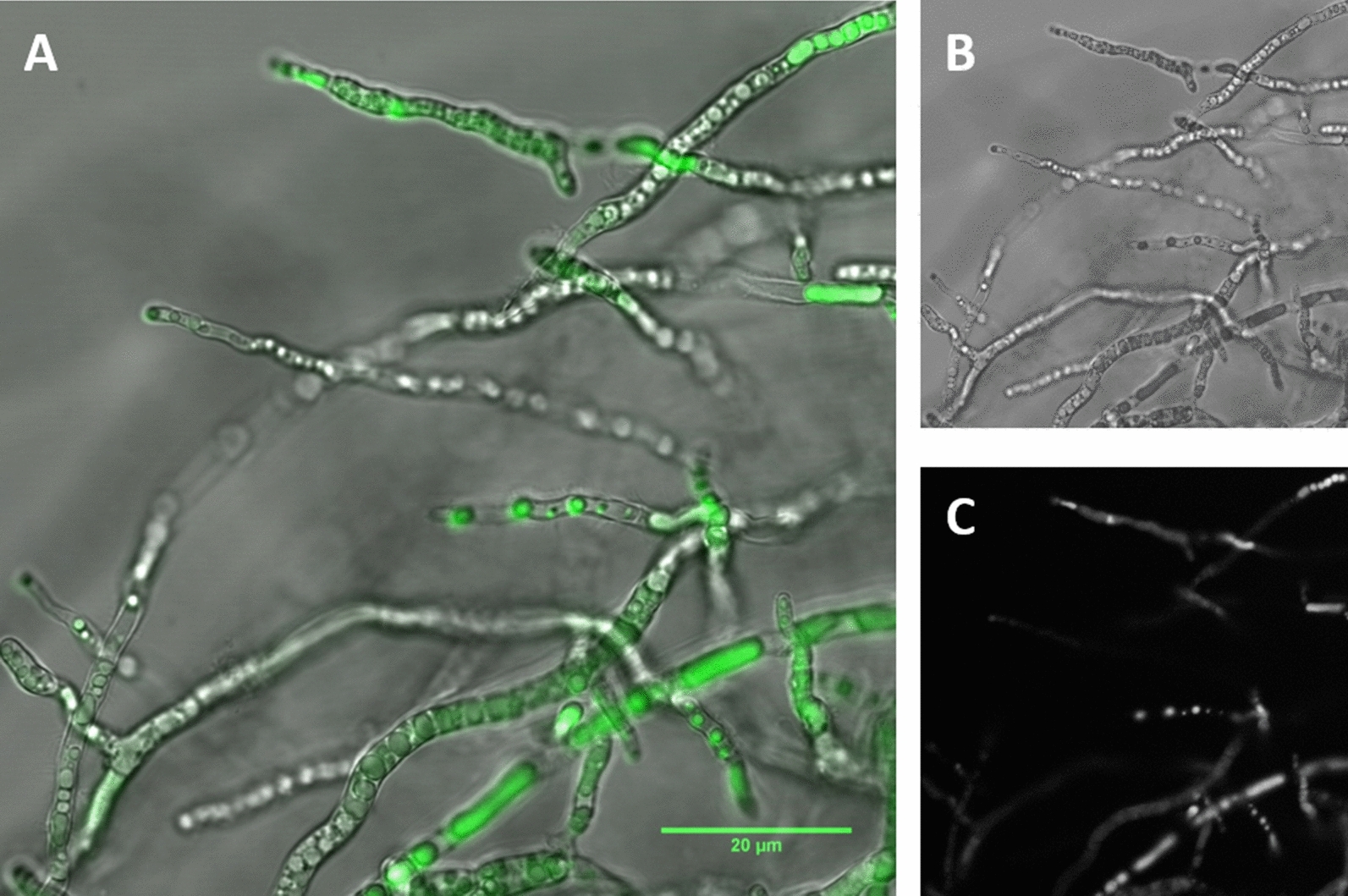
Overlayed confocal laser scanning micrograph images **A** of accumulated xanthoepocin (green fluorescent) in hyphae of ammonium limited grown bioreactor batch cultures of *Pencillium ochrochloron* CBS123823 after 90 h of cultivation at ambient light. **B** Brightfield channel, **C** Fluorescence channel (λ_exc_/λ_em_ = 395/430 nm)

As the (photo)chemical characterization showed that xanthoepocin is fluorescent (see “(Photo)chemical characterization of xanthoepocin” Section), hyphae from later stages of the cultivation were additionally examined with laser confocal microscopy. It revealed that the metabolite is concentrated within vacuoles, which are distributed all over the hyphae (Fig. 7).

### Antibacterial susceptibility tests

The estimation of the minimal inhibitory concentrations (MIC) of xanthoepocin and tigecycline was performed according to the standards of the Clinical and Laboratory Standards Institute (CLSI, 2012). The susceptibility for both antibiotics was tested with *Escherichia coli* ATCC 25922, *Staphylococcus aureus* ATCC 29213, a methicillin-resistant *Staphylococcus aureus* strain, and a *Enterococcus faecium* strain with a combined linezolid and vancomycin resistance.

#### MIC test of tigecycline

For *S. aureus* ATCC 29213, the methicillin-resistant *S. aureus* strain, and *E. coli* ATCC 25922, the evaluated MIC of tigecycline was 0.156 µg mL^−1^ (c = 0.27 µM). In the linezolid and vancomycin-resistant *E. faecium* strain, growth inhibition occurred at a tigecycline concentration between 0.156 and 0.313 µg mL^−1^ (c = 0.27–0.53 µM). The control for testing a potential solvent interferences with DMSO (1:10, v/v without tigecycline) showed no inhibitory or promotional effect on bacterial growth.

#### MIC test of xanthoepocin

Growth inhibition occurred at a concentration of 0.313 µg mL^−1^ (c = 0.52 µM) in the case of *S. aureus* ATCC 29213 and the methicillin-resistant *S. aureus* strain, whereas xanthoepocin showed no effect on *E. coli* ATCC 25922 at any concentration. Growth inhibition of the *E. faecium* strain with a combined resistance against linezolid and vancomycin was detected at a xanthoepocin concentration between 0.078 and 0.313 µg mL^−1^ (c = 0.13–0.52 µM).

### Photoantimicrobial susceptibility test

Photoantimicrobial assays were carried out employing a recently established and validated irradiation protocol [30]. In detail, after ten minutes of incubation, the treated bacteria suspension was irradiated with blue light (λ = 428 ± 15 nm). Two different light doses (H = 9.3 J cm^−2^ and H = 30 J cm^−2^) were applied in two independent experiments and correlated to the respective, analogous experiment which was kept in the dark. Against *E. coli*, the light irradiation had no additional effect. Against *S. aureus*, however, a clear impact was observed: Light irradiation abolished the effect of xanthoepocin (Additional File 1: Fig. S21). While in the dark, a MIC of 0.3125 µg mL^−1^ (c = 0.52 µM) was also determined against the strain DSM1104 of *S. aureus*, blue light irradiation led dose-dependent to a PhotoMIC of either 2.5 µg mL^−1^ (c = 4.16 µM, H = 9.3 J cm^−2^) or above 5 µg mL^−1^ (c > 8.32 µM, H = 30 J cm^−2^).

## Discussion

*Pencillium ochrochloron* has been the primary model organism of our workgroup for many decades. We frequently observed that bioreactor batch culture broths of one strain, *P. ochrochloron* CBS123823, turned yellowish under certain conditions. In the course of a re-identification [31], a secondary metabolite profiling identified the yellow pigment xanthoepocin as one of the compounds produced (Jens Frisvad, personal communication). Although xanthoepocin is a widespread metabolite within the genus Penicillium, the trigger factors leading to its biosynthesis or the biological function are still unknown. However, its reported bioactivity against methicillin-resistant *Staphylococcus aureus* (MRSA) [11] and the fact that it entered a preclinical test phase [12] make this metabolite highly interesting in terms of a re-investigation.

Thus, the initial aim of our work was (i) to explore the physiological prerequisites triggering the production of this metabolite, (ii) to isolate larger quantities of xanthoepocin to develop a suitable routine quantification method, and (iii) to perform an in-depth chemical characterisation that goes beyond the initial study of Igarashi et al. [11]. (iv) Last but not least, we were interested if the reported bioactivity against MRSA [11] can be verified and if xanthoepocin is also active against multiresistant *Entercoccus faecium*, which is one of the leading causes of nosocomial infections [32]. For the latter, we used a clinical isolate of *E. faecium* with a combined linezolid and vancomycin resistance (LVRE)—a problematic multi-resistance that is currently on the rise [32].

### Improved isolation and (photo)chemical characterisation

In a first step, xanthoepocin was isolated as described by Igarashi et al. [11] and characterized by state-of-the-art techniques, confirming the successful isolation of xanthoepocin. Additionally, during the isolation and characterization process, we gained new information about (i) the light sensibility, (ii) the acidic instability, and (iii) the aggregation behaviour of this metabolite.

Based on the observed light and acidic instability, the isolation process was optimized. In short, light exposure and total retention time on silica were minimized by utilizing vacuum filtration chromatography on silica followed by a semipreparative and preparative column chromatography on reverse phase material. Following this protocol, a total yield of η = 1.5% calculated from the dry weight of the mycelium was achieved, and thus an improvement by factor 50, as compared to our non-optimized protocol.

With more material in hand, a photochemical characterization was possible, proving the light instability of xanthoepocin under blue light irradiation (Fig. 2B, Additional file 1: Fig. S9). Photochemical and photophysical methods proved that xanthoepocin is a photosensitizer and can be characterized by a photochemical singlet oxygen production quantum yield of Φ_Δ_ = 7.8% in deuterated methanol (Additional file 1: Fig. S10B). Compared to the photochemical singlet oxygen production quantum yield of other polyketides from fungi it can be stated that xanthoepocin is not as photoactive as conjugated bisanthrons like cercosporin (**5**), elisonchrome A, or hypocrellin B (i.e., ϕ_Δ_ = 81%, 94%, and 83%, respectively) [33]. However, compared to other dimeric polyketides like (S)-5,5’-bisoranjidiol [34] (ϕ_Δ_ = 18%), the photoactivity is in a more similar range.

### Factors triggering xanthoepocin biosynthesis

As mentioned before, we frequently observed that bioreactor batch cultivations of the wild-type strain *P. ochrochloron* CBS 123823 exhibited yellowish culture broths, especially in ammonium- and phosphate-limited media (unpublished results). These nutrient limitations are often reported to trigger pigment production in filamentous fungi [35]. Since xanthoepocin showed a distinctive light instability (see “Effect of light on Xanthoepocin” Section), we hypothesized that its production in *P. ochrochloron* CBS 123823 is possibly favored under ammonium- and/or phosphate-limited conditions combined with darkness.

To test this hypothesis, we started a series of explorative petri dish experiments with different defined nutrient-limited media and variations in the irradiation conditions, which caused vast changes in the phenotype of *P. *ochrochloron (Fig. 3). The exploratory experiment highlighted ammonium-limitation as the most favorable nutrient limitation for xanthoepocin production combined with longer wavelengths or darkness (Fig. 4). The same tendency was found in ammonium-limited bioreactor batch cultures: The xanthoepocin content was drastically reduced under blue light illumination and fostered under red light or darkness (Fig. 6). A laser confocal microscopic study revealed that xanthoepocin is a pigment that is intracellularly concentrated in vacuoles (Fig. 7).

Nutrient-dependent light responses such as these are scarcely explored in fungi with defined media. One reason for this is that undefined complex standard media are often preferred to minimal media in many microbiological experiments. In consequence, the physiological state of the culture often remains unknown and thus severely limits the interpretation of the results [36]. However, the few available data on this issue underline that there is a tight interplay between nutritional state, light conditions, and physiological response [27]. Also, the synthesis of many fungal pigments, including polyketides such as xanthoepocin, is dependent on the interplay between nutritional and light conditions [35]. Of particular interest in this context is the red polyketide pigment cercosporin (Fig. 1, **5**) produced by several plant pathogenic fungi such as *Alternaria alternata* and fungi of the genus *Cercospora* [37, 38]. Cercosporin is structurally related to xanthoepocin and a potent photosensitizer in the presence of light [37, 38], but in contrast to xanthoepocin, its production is repressed in darkness and under nutritional conditions which foster conidiation [37]. Despite the wealth of data on factors influencing fungal polyketide pigment production, a profound understanding of the underlying regulatory mechanisms is still missing since the involved metabolic pathways are very complex [35]. However, an in-depth understanding of these mechanisms is needed to meet the increasing demand for fungal pigments in various industrial fields [39, 40].

### (Photo)antibacterial susceptibility test

In 2018, the WHO declared methicillin-resistant *Staphylococcus aureus* (MRSA) and vancomycin-resistant *Enterococcus faecium* (VRE) as the two high-priority gram-positive bacteria for which novel therapeutics should be developed [41]. Especially treating VRE infections is a considerable problem since against these bacteria only a handful of therapeutics are available [42, 43]. These comprise the tetracycline tigecycline, the lipopeptide daptomycin, and the oxazolidinones tedizolid and linezolid [43]. However, the recent rise of infections with a combined linezolid and vancomycin resistance (LVRE) [32] limits the therapeutic options against these bacteria even more [42].

Since previous research [11] indicated that xanthoepocin is significantly bioactive against gram-positive bacteria and even MRSA, we chose clinical isolates of MRSA and LVRE amongst other organisms to test its effectiveness. The tetracycline tigecycline was used as a control.

Xanthoepocin did not affect the growth of the gram-negative bacterium *E. coli* ATCC 25922, at least in the tested concentrations (i.e., up to 10 µg mL^−1^, 16.52 µM; darkness), but was active against all gram-positive bacterial strains tested in this study. While the resulting MIC values for tigecycline against *S. aureus* ATCC 29213 (0.156 µg mL^−1^; c = 0.27 µM; darkness) and a methicillin-resistant *S. aureus* strain (0.156 µg mL^−1^; c = 0.27 µM; darkness) were in the range of previously reported MIC values [44], the MIC values estimated for xanthoepocin in this study (0.313 µg mL^−1^; c = 0.52 µM; darkness) were approximately fivefold lower than the ones previously published (1.56 µg mL^−1^; c = 2.58 µM; uncontrolled lightning conditions) [11]. These lower MIC values might be explained either by a higher susceptibility of our test-strains towards xanthoepocin, or are –more likely– caused by the applied light-protecting measures, which prevented the metabolite from degrading. Indeed, a photoantimicrobial assay exploring a potential photopharmacological use of xanthoepocin, corroborated this hypothesis as the loss of activity was light-dose dependent (Additional file 1: Fig. S21) and correlated with the decay of this metabolite.

The determined MIC values for tigecycline against a clinical LVRE isolate were in the range of 0.156–0.313 µg mL^−1^ (c = 0.27–0.53 µM). The higher MIC value already indicates a resistance according to the 2021 EUCAST definition of resistance for this antibiotic [45], i.e., MIC > 0.25 µg mL^−1^ (c = 0.43 µM). Against the same strain, we estimated MIC values for xanthoepocin in the range of 0.078–0.313 µg mL^−1^ (c = 0.13–0.52 µM) in the dark, which is an interesting range of concentration for a potential medical application.

Taken together, xanthoepocin was highly active against the two top high-priority multiresistant gram-positive bacteria listed recently by the WHO [41]. Due to the light protection measures, we were even able to achieve fivefold lower MIC values against MRSA than previously published [11]. We, therefore, conclude that a further reexamination of xanthoepocin is of high interest.

## Conclusions

In this work, we could underline that the proposed reexamination of known compounds with state-of-the-art-techniques is an important avenue in the combat against multiresistant bacteria. With appropriate light protecting measures, the obtained MIC values for xanthoepocin significantly surpassed published values, thus proving this metabolite to be a powerful antibiotic against MRSA and LVRE. Considering how widespread similarly structured polyketides are within nature, an in-depth re-evaluation of this metabolite class with respect to its photo-responsiveness is of utmost relevance. There is a high likelihood that formally discarded aromatic polyketides could be re-introduced into the antibiotic pipeline with appropriate light protecting measures or find application as a photosensitizer for (antimicrobial) photodynamic therapies.

The presence of light was, besides the nutritional state of the hyphae, a decisive parameter for the xanthoepocin content in *Penicillium* *ochrochloron*. In this work, we could show for the first time that intracellular xanthoepocin concentrations were the highest under ammonium-limited conditions in combination with irradiation wavelengths above 519 nm or in darkness. Whether or not the drastic decrease of the intracellular xanthoepocin content under blue light irradiation is a direct consequence of its degradation or a result of the suppression of its biosynthesis is still an open question. Also, in an ecological context, the multifaceted light-dependency of xanthoepocin is of interest, as this metabolite is widely spread amongst the genus Penicillium and must pose a particular evolutionary advantage.

In the interdisciplinary context of this study, light turned out to be a pivotal factor that influenced the majority of performed experiments ranging from the isolation process and the analytics, the cultivation of the organism, and the susceptibility test. As an interdisciplinary team, we thus conclude that it is essential for the progress of the field to pay more attention to light as an experimental parameter.

## Methods

### Chemicals and organisms

#### Chemicals

All media components for the cultivations with *Penicillium ochrochloron* were purchased from Merck (Darmstadt, Germany) with the exception of HEPES (Roth, Darmstadt, Germany). The reference antibiotic tigecycline and the 96-well plates were purchased from Sigma Aldrich (St. Louis, Missouri, USA). Antibacterial susceptibility testing was performed with Mueller Hinton Bouillon (MHB, Becton Dickinson, New Jersey, USA) as a medium. All solvents were obtained from VWR International (Vienna, Austria). Ethyl acetate and acetone were additionally distilled according to the ÖAB (Österreichisches Arzneibuch). Ultra pure water was obtained utilizing the Sartorius arium® 611 UV purification system (Sartorius AG, Göttingen, Germany). Silica gel 40–63 μm and pre‐packed cartridges for flash chromatography were purchased from Merck (Darmstadt, Germany) and Büchi (BÜCHI Labortechnik AG, Flawil, Switzerland), respectively. Thin-layer chromatographic analysis was performed using silica TLC plates 60 F254 (Merck, Darmstadt, Germany).

#### Organisms

Petri dish and bioreactor batch experiments were performed with *Penicillium ochrochloron* CBS 123823. The bacteria used for antimicrobial testing were *Staphylococcus aureus* ATCC 29213 and *Escherichia coli* ATCC 25922 obtained from the American Type Culture Collection (ATCC®, Virginia, USA) as well as a methicillin-resistant *Staphylococcus aureus* strain (MRSA, clinical isolate, wild-type), and *Enterococcus faecium* strain resistant to linezolid and vancomycin (LVRE, clinical isolates, wild-type). For photoantimicrobial assays, *E. coli* (DSM1103, DSMZ-German Collection of Microorganisms and Cell Cultures, Braunschweig, Germany) and *S. aureus* DSM1104 (DSMZ-German Collection of Microorganisms and Cell Cultures, Braunschweig, Germany) were used*.*

### (Photo)Chemical characterisation of xanthoepocin

#### Instruments

For the isolation process the following instruments were utilized: The mill MF10 basic (IKA Labortechnik, IKA®-Werke GmbH & Co. KG, Staufen, Germany), the balances KERN ALS 220-4 (KERN & SOHN GmbH, Balingen-Frommern, Germany) and Sartorius Cubis®-series (Sartorius AG, Göttingen, Germany), the rotary evaporators Heidolph LABOROTA 4000-efficient, Heidolph Hei-VAP Precision (Heidolph Instruments GmbH & CO. KG, Schwabach, Germany), and IKA RV 10 (IKA®-Werke GmbH & Co. KG, Staufen, Germany). Furthermore, the ultrasonic bathes Sonorex RK 106, Sonorex RK 52, and Sonorex TK 52 (BANDELIN electronic GmbH & Co. KG, Berlin, Germany) were utilized and the flash-chromatography system Reveleris® X2 (BÜCHI Labortechnik AG, Flawil, Switzerland). HPLC measurements were carried out using the modular system Agilent Technologies 1260 Infinity II and the Agilent Technologies 1200 Series from Agilent Technologies, Inc. (Santa Clara, USA). For preparative HPLC chromatography, a Dionex (Thermo Fisher Scientific Inc., Waltham, USA) HPLC-system, consisting of a Dionex UltiMate 3000 pump, an ASi-100 Automated Sample Injector, a Dionex UltiMate 3000 Column Compartment, a Dionex UVD170U, and a Gilson 206 Fraction collector in combination with a Synergi MAX-RP 80 Å column (250 × 10.00 mm, 4 micron) from Phenomenex (Aschaffenburg, Germany) was used. NMR spectra were acquired using two spectrometers from Bruker, an Avance II 600 spectrometer operating at 600 MHz (^1^H) and 151 MHz (^13^C) and an Avance III HD spectrometer operating at 400 MHz (^1^H) (Bruker Corporation, Billerica, USA). The spectra were recorded in deuterated solvents supplied by Euriso-Top (Cambridge Isotope Laboratories, Inc., Saint-Aubin, France). Optical rotation data was acquired on a JASCO P-2000 polarimeter (Jasco Deutschland GmbH, Pfungstadt, Germany). IR spectra were recorded on an ALPHA FT-IR apparatus (Bruker, Ettlingen, Germany) equipped with a Platinum ATR module.

#### Isolation of xanthoepocin

The dried bioreactor batch mycelium (see also “Submerged cultures—bioreactor batch experiments” Section) was fine-milled with an electric mill (MF10 basic, IKA, Germany) at 3750 rpm to the smallest grain size and transferred (60 g) into an Erlenmeyer flask (500 mL) covered with aluminum foil. The mycelium was first defatted with petroleum ether (3 × 200 mL, 30 min ultrasonication each) and afterward extracted with acetone (3 × 200 mL, 30 min sonication each). The acetone fractions were combined and reduced under vacuum. The resulting ochrous, viscose liquid was mixed with C18 material and submitted to flash chromatography (Reveleris®) using a C18 column (40 µm, 80 g). As the mobile phase, acidified water (H_2_O + 0.1% HCOOH) and acetonitrile (ACN + 0.1% HCOOH) were used. As gradient, the following system was used: t = 0 min, 10% B–> t = 10 min, 90% B–> t = 30 min, 90%B. The blue light-absorbing fractions were combined, reduced, and yielded a dark yellow solid, xanthoepocin (m = 142.6 mg). Further purification was done via preparative HPLC employing a MaxRP 80 Å (4 µm, 850 × 10 mm) column from Synergy (Phenomenex, Aschaffenburg, Germany). The following gradient system was employed: t = 0 min, 10% B –> t = 7.5 min, 90% B –> t = 20 min, 90% B. As mobile phase, water and acetonitrile were used. During the entire process, light was excluded as much as possible.

#### HPLC analysis of the extracts

The concentrated extract derived either from extracted cultures of the petri dish experiment (see “Solid state cultures—petri dish experiments” Section), or the bioreactor experiment (see “Submerged cultures—bioreactor batch experiments” Section) was solved in DMSO (V = 1 mL), filtered (PFTE, 0.45 µm), and submitted to an HPLC–DAD analysis utilizing a Synergi MaxRP (80 Å, 150 × 4.60 mm) column and water as well as acidified acetonitrile (ACN + 0.1% HCOOH) as mobile phase. The gradient started at 50% B, was increased to 85% in the first 7.5 min, and further increased to 98% B until t = 20 min. A needle wash and a post and pre-run washing step (5 and 10 min, respectively) was implemented between the measurements. The DAD chromatogram (λ = 396 nm) was extracted and integrated via Origin 2020 (OriginLab Corp., Northampton, USA).

#### Dynamic light scattering experiments

A stock solution of xanthoepocin (5 mM, DMSO) was prepared and diluted with MHB medium (1:10 and 1: 1000 yielding a c = 500 µM and c = 5 µM solution). The size distribution was measured at T = 25 °C. The medium without analyte was analyzed as blank.

To determine the critical aggregation concentration (CAC) the stock solution was diluted in PBS and the count rate was measured in triplicates at T = 25 °C. The average of each count rate was corrected by the attenuation factor and plotted against the concentration. Linear fitting was done with Origin 2020 (OriginLab Corp., Northampton, USA).

#### DMA assay

The DMA assay was performed utilizing 9,10-dimethylanthrance (DMA) solubilized as chemical probe in ethanol as previously described [26]. In short, xanthoepocin was solved in DMSO and diluted with ethanol. The chemical probe DMA, or the probe with ascorbic acid, or ascorbic acid alone (c = 1 g mL^−1^) were added as well. As a reference, berberine (c = 1 mg mL^−1^, 2.97 mM, DMSO, V = 10 µL) was used. Before and after each irradiation step (5 cycles, each λ_irr_ = 468 nm, H = 1.24 J cm^−2^ min^−1^, t = 5 min) the optical densities at λ = 377 nm and λ = 468 nm were recorded as technical duplicate. The observed difference in the DMA absorbance was determined for each sample set.

#### Emission and absorbance measurements

A xanthoepocin solution was prepared in deuterated methanol, and the absorbance coefficient calculated from five concentrations (below 150 µM). To guarantee an equal distribution, each solution was filtered (400 µm) immediately before the spectroscopic measurement. A xanthoepocin solution in methanol was prepared with an OD of approximately 0.1 at the detection wavelength of λ = 450 nm. The visible light emission of xanthoepocin and the near-infrared emission of singlet oxygen was detected at λ = 1270 nm as previously reported [46]. An absorbance measurement (200–800 nm) before and after excitation was recorded to assure the stability of xanthoepocin. As an excitation source, a 450 nm CW laser diode was utilized (0.4 W/cm^2^). A solution of [Ru(bpy)_3_]Cl_2_ in deuterated methanol served as reference (Φ_Δ_ = 0.73).

#### Decomposition studies

Initially, a solution of xanthoepocin was placed at the window and submitted to a HPLC measurement after t = 5 min and ten days. Having observed considerable effects of sunlight already after five minutes, a more thorough experiment was conducted: A solution of xanthoepocin in d_6_ DMSO (39.3 mg mL^−1^) was irradiatated with blue light (λ = 455 ± 30 nm, 0.64 mW cm^−2^) in a 96 well plate for 20 min. Samples for the HPLC-analysis (V = 200 µL) were withdrawn every minute for the first five minutes, then every second minute until 10 min, and finally after 15 and 20 min. The samples were anylzed by HPLC–DAD with the established method (see HPLC analysis).

#### Light stability measurement

Light stability of xanthoepocin was measured in deionized water. A xanthoepocin stock solution (c = 1 mg mL^−1^) was prepared. A part of the stock solution (V = 0.30 mL) was diluted with water (V = 9.7 mL) to a final concentration of c = 30 µg mL^−1^. The sample (final xanthoepocin concentration c = 49 µM) was deoxygenated with argon for 20 min prior to the measurements. The samples were stirred under argon and kept at 297 K for the duration of the experiment. Irradiation was performed using a custom 413 nm LED (0.86 mW, 95 min, 5 J cm^−2^), and UV–Vis absorbance spectra were measured every 30 s. In parallel, an identical solution was studied in the dark.

### Solid state cultures—petri dish experiments

#### Media

To perform the explorative screening experiments, agar plates with 20 mL of either a glucose-limited, ammonium-limited, or phosphate-limited medium were prepared according to Additional file 1: Table S1. Glucose and salt solutions were autoclaved separately to avoid the formation of toxic compounds. All media were set to pH 7 before autoclaving and were combined under sterile conditions after cooling down to room temperature. The trace element solution (composition see [31]) was added sterile filtered.

#### Cultivation and illumination conditions

For the screening experiment, each petri dish was inoculated three times with a needle, which was dipped into a spore suspension with a spore density of 5 × 10^8^ spores. Afterward, each inoculated petri dish was placed in separate light-impermeable carton boxes (Additional file 1: Fig. S12) and continuously irradiated with one light-emitting diode (LED; Additional file 1: Fig. S13A) for seven days in a climate chamber at 25 °C. Spectrum (Additional file 1: Fig. S13B) and intensity (Additional file 1: Table S2) of the illumination conditions was determined with a radiometer (Thorlabs PM100D, Silicon Power Head 200 nm–1100 nm) and a spectrometer (Ocean Optics Maya 2000 Pro, Grating HC-1 200 nm to 1050 nm, Optical Fiber 600 µm), respectively. After seven days of incubation, the cultures were photo-documented, and the colony diameters were determined before the colonies were extracted.

#### Xanthoepocin extraction of fungal colonies from solid media

Variations in colony morphology, (i.e., glucose-limited grown cultures grew flat and loose, whereas ammonium- and phosphate-limited cultures grew dense and slightly elevated), made it necessary to adjust the way the colonies were detached from the agar plates. Glucose-limited grown colonies were cautiously scratched from the surface of the agar plate with a scalpel. This was not possible with ammonium- or phosphate-limited colonies, which had to be excised from the agar plate with the scalpel and any agar remnants carefully removed afterward. Independent of the medium, the single colonies were then transferred to 10 mL amber-colored glass vials, and 5 mL acetone was added for xanthoepocin extraction. For additional light protection during the extraction step, the vials were covered with aluminium foil and ultrasonicated for 15 min. Afterward, the extract was filtered through a cotton-filled Pasteur pipette into a second amber-colored glass vial, which was also covered in aluminium foil. Before the subsequent evaporation step, the foil was permeated with a needle. The acetone was evaporated in a constant air stream overnight until only the pure extract remained in the vial.

With this method, the whole colony had to be extracted, and thus the obtained xanthoepocin values could not be attributed to a normalized biomass value. Nevertheless, the obtained data allowed valuable first insights into a potential nutrient- and light-dependent xanthoepocin production since ammonium- and phosphate-limited grown cultures showed cultures with comparable sizes (Additional file 1: Fig. S14) and the xanthoepocin levels of glucose-limited cultures were significantly lower (Fig. 2).

### Submerged cultures—bioreactor batch experiments

#### Preculture and bioreactor batch medium

To obtain a filamentous mycelium in the preculture, *P. ochrochloron* was cultivated in a Glucose-HEPES medium with 1 M HEPES (pH 7.3) for 72 h at 30 °C on a rotary shaker at 350 rpm as described earlier [29]. Each bioreactor batch cultivation was inoculated with three precultures.

Since the screening experiments revealed that the xanthoepocin content was highest under ammonium-limited conditions, this nutrient limitation was chosen for the bioreactor batch experiments. Similar to the petri dish experiments, glucose and salt solutions (composition ammonium limited medium see [29]) were autoclaved separately and combined under sterile conditions after reaching room temperature. 10 mL trace element solution per liter medium (composition see [31]) was filtrated (0.2 µm) and added under aseptic conditions.

#### Cultivation in bioreactors and illumination conditions

All bioreactor batch experiments were performed in a Biostat B bioreactor (Braun Sartorius, Germany) with a working volume of 4.6 L at 25 °C, 890 rpm, and at an aeration rate of 0.56 vvm as previously described [29]). The pH was kept constant at pH 7 with sterile 5 M NaOH.

The bioreactor batch experiments were performed with four individual illumination settings, meaning three different irradiation scenarios and one cultivation in the absence of light as control. Each light setting varied in intensity and spectral composition, as it is shown in Additional file 1: Fig. S16. White light conditions were realized with the existing laboratory light (fluorescent tubes, Philips TLD 18 W/33–640). Blue light together with red light conditions were achieved with single color high flux light-emitting diodes (blue: Osram Opto Semiconductors LD CQDP-2U3U-W5-1 and red: Osram Opto Semiconductors LH CPDP-2T3T-1–0). Details of the used light sources and prevailed intensities on the outside of the bioreactor vessel are summarized in the Additional file 1: Table S3.

To ensure constant irradiation conditions, an efficient thermal management of the LEDs was necessary. Therefore, the LEDs were placed on an aluminum core circuit board to guarantee sufficient heat dissipation. To avoid any unwanted light contamination, the bioreactor vessel was covered with fabrics impermeable to light (Additional file 1: Fig. S16A). Light intensity was measured with a radiometer (Thorlabs PM100D, Silicon Power Head 200 nm–1100 nm, wavelength settings according to weighted response curve were 451 nm, 570 nm, and 660 nm for blue, white, and red light conditions, respectively) at different positions outside the vessel and was determined as the mean value of each measurement point. The spectral composition was gained with a modular spectrometer (Ocean Optics Maya 2000 Pro, Grating HC-1 200 nm to 1050 nm, Optical Fiber 600 µm). The corresponding values of the photon flux density were calculated whilst taking the spectral composition of the light source into account.

#### Sampling and harvesting

The bioreactors were sampled regularly, whereby for each sampling time point, triplicate samples were withdrawn from the bioreactor. The triplicate samples (5 mL each) were filtered with a vacuum pump (Millipore, Darmstadt, Germany) through a preweighed cellulose acetate filter (Braun Sartorius, Germany) with a pore size of 0.45 µm. Afterward, the obtained filtrate was aliquoted into 1.5 mL tubes and stored at -20 °C until later nutrient analytics. In addition to the triplicate samples, 10–15 mL of the culture broth was filled in 15 mL falcons and also stored at -20 °C until later analysis. Residual nutrient concentrations were measured as described elsewhere [47]. The filters with the biomass were dried at 105 °C to estimate the dry weight.

After five days of cultivation (approximately 92 h), the bioreactor batch cultivations were stopped. The culture broth was harvested and filtered through a dish towel or fabric sheet into a bucket. The resulting mycelium was wrung by hand to remove most liquid, then laid out flat on a tablet and dried at 40 °C overnight. The dried mycelium and the filtrate were stored separately at -20 °C until further use.

#### Xanthoepocin analytic of dried mycelia

The dried mycelium was ground with an electric mill (MF10 basic, IKA, Germany) at 3750 rpm to the smallest grain size before it was sieved to 0.5 mm, which provided a homogeneous grain size. Approximately 25 mg (exact weight noted) of the ground mycelium was filled in 1.5 mL amber-colored eppendorf tubes in triplicates.

To each tube, 1 mL acetone was added. In the next step, all tubes were exposed to ultrasonication for 15 min and then centrifuged (5804 R, Eppendorf, Germany) at 14,000 rpm for 10 min at 4 °C. The resulting supernatant, which contained xanthoepocin, was decanted into amber-colored glass vials and covered with perforated aluminum foil. This ultrasonic-assisted acetone extraction was again repeated twice with the mycelial pellet in the tubes. All three resulting supernatants of one mycelium were collected in the same glass vial (i.e., each vial contained eventually 3 mL acetone extract) before it was placed under an air stream until the acetone was evaporated entirely.

#### Photo-documentation of the culture broth

To photo-document pigmental changes during the cultivation process, the culture broth of each sampling time point was thawed, and one milliliter was transferred into micro cuvettes. The micro cuvettes of each bioreactor batch experiment were then arranged in the order of the sampling times and photographed in a standardized setting.

#### Confocal microscopy

A suspension of *P. ochrochloron* in PBS was analyzed under the confocal microscope (Leica TCS SPE5 II) utilizing an excitation beam fitting the absorbance range (i.e. λ =  ± 390 nm) and a emission filter fitting the emission range (i.e. λ =  ± 516 nm) of xanthoepocin respectively. An 40 × magnification was utilized, and the pictures were taken shortly after focusing the sample to avoid a photobiological reaction.

### Antibacterial susceptibility tests

All bacteria for susceptibility testing were grown overnight on BD Columbia Agar plates (Becton Dickinson, New Jersey, USA) at 37 °C.

The evaluation of the MIC of xanthoepocin and tigecycline was performed by a serial dilution according to the Clinical and Laboratory Standards Institute (CLSI, 2012), which defines the MIC as the lowest concentration of a compound leading to a visible growth inhibition after overnight incubation. For this purpose, all tested bacterial cultures were adjusted to 0.5 McFarland standard turbidity in saline, which corresponds to a bacterial suspension with a concentration of 1.5 × 10^8^ colony-forming units per milliliter (CFU mL^−1^). Subsequently, 50 µl of the bacterial suspension was added to 10 mL MHB.

The antibacterial activity of xanthoepocin was tested against the antibiotic tigecycline. The preparation of the stock solutions was done by dissolving xanthoepocin or tigecycline in DMSO at a concentration of 1 mg mL^−1^ and subsequent 1:10 dilution in sterile water. Afterward, a serial dilution was conducted in a flat-bottomed 96-well tissue plate with concentrations ranging from 10 to 0.01 µg mL^−1^. After the addition of 90 µl of the bacterial suspension into each well, the plates were incubated at 35 ± 2 °C overnight.

Prior to the determination of the optical density (OD), the plates were shaken at 250 rpm for 15 min. The OD was measured at 490 nm using the Bio-Rad 680 microplate reader (Hercules, California, USA). Growth inhibition in the presence of xanthoepocin and tigecycline, respectively, was assumed in case growth was reduced for at least 5 log units. Diluted DMSO (1:10, v/v) without xanthoepocin or tigecycline, respectively, served as growth control.

All procedures for bacterial susceptibility testing were performed twice under analogous conditions for each bacterial strain.

### Photoantimicrobial susceptibility testing

The photoantimicrobial experiments were carried out as described previously [30]. In brief, a dilution series of xanthoepocin was prepared in MHB according to the CLSI guidelines (CLSI, 2012), and transferred into two identical 96-well plates. The 96-well plates for the microdilution tests were always prepared freshly. The inoculum was prepared from an overnight culture of *S. aureus* (DSM1104) or *E. coli* (DSM1103) and adjusted to a McFarland standard of 0.5 via turbidity measurement (λ = 600 nm). After dilution by 1:100 with MHB, 50 µL of inoculum was given to 100 µl medium and 50 µl of diluted fraction into each well.

One plate served as dark condition control. After ten minutes of incubation, the second plate was irradiated with blue light (λ = 428 ± 15 nm, H = 30 J cm^−2^ or 9.3 J cm^−2^). Afterward, the plates were incubated at T = 37 °C in the dark for 24 h. Assessment of the experiment was done by correlating the turbidity of the treated wells to the uninhibited growth control. Irradiated and dark samples were evaluated separately.

## Supplementary Information

**Additional file 1. **Additional tables and and figures.

## Data Availability

The datasets supporting the conclusions of this article are included within the article and its additional files.

## Abbreviations

η: Yield
Φ_Δ_: Photoyield
λ: Wave length
ACN: Acetonitrile
CAC: Critical aggregation concentration
DAD: Diode array detection
DMA-assay: 9,10-Dimethylanthracen assay
DMSO: Dimethyl sulfoxide
HCOOH: Formic acid
HEPES: 4-(2-Hydroxyethyl)-1-piperazineethanesulfonic acid
HPLC: High pressure liquid chromatography
LED: Light emitting diode
LOD: Limit of detection
LQ: Limit of quantification
LVRE: Linezolid and vancomycin-resistant *Enterococcus faecium*
m: Mass
NaOH: Sodium hydroxide
MIC: Minimal inhibitory concentration
MHB: Müller Hinton broth
MRSA: Methicillin-resistant *Staphylococcus aureus*
MS: Mass spectrometry
NMR: Nuclear magnetic resonance
OD: Optical density
PBS: Phosphate-buffered saline
(a)PDT: (Antimicrobial) photodynamic therapies
ROS: Reactive oxygen species

## References

[1] O'Neill: Tackling Drug Resistant Infections Globally: final report and recommendations. https://amr-review.org/sites/default/files/160518_Finalpaper_withcover.pdf. Accessed 15 Dec 2021.

[2] Donadio S, Maffioli S, Monciardini P, Sosio M, Jabes D (2010). Sources of novel antibiotics-aside the common roads. Appl Microbiol Biotechnol.

[3] Nass NM, Farooque S, Hind C, Wand ME, Randall CP, Sutton JM, Seipke RF, Rayner CM, O'Neill AJ (2017). Revisiting unexploited antibiotics in search of new antibacterial drug candidates: the case of gamma-actinorhodin. Sci Rep.

[4] Wright GD (2017). Opportunities for natural products in 21st century antibiotic discovery. Nat Prod Rep.

[5] Sonjak S, Licen M, Frisvad JC, Gunde-Cimerman N (2011). The mycobiota of three dry-cured meat products from Slovenia. Food Microbiol.

[6] Houbraken J, Lopez-Quintero CA, Frisvad JC, Boekhout T, Theelen B, Franco-Molano AE, Samson RA (2011). *Penicillium araracuarense* sp. nov., *Penicillium elleniae* sp. nov., *Penicillium penarojense* sp. nov., *Penicillium vanderhammenii* sp. nov. and *Penicillium wotroi* sp. nov., isolated from leaf litter. Int J Syst Evol Microbiol.

[7] Kildgaard S, Mansson M, Dosen I, Klitgaard A, Frisvad J, Larsen T, Nielsen K (2014). Accurate dereplication of bioactive secondary metabolites from marine-derived fungi by UHPLC-DAD-QTOFMS and a MS/HRMS library. Mar Drugs.

[8] Taniwaki MH, Pitt JI, Iamanaka BT, Massi FP, Fungaro MHP, Frisvad JC (2015). *Penicillium excelsum* sp. nov from the Brazil nut tree ecosystem in the Amazon Basin’. PLoS ONE.

[9] Grijseels S, Nielsen JC, Randelovic M, Nielsen J, Nielsen KF, Workman M, Frisvad JC (2016). *Penicillium arizonense*, a new, genome sequenced fungal species, reveals a high chemical diversity in secreted metabolites. Sci Rep.

[10] Teixeira MF, Martins MS, Da Silva JC, Kirsch LS, Fernandes OC, Carneiro AL, Da Conti R, Durán N (2012). Amazonian biodiversity: pigments from aspergillus and penicillium-characterizations, antibacterial activities and their toxicities. Curr Trends Biotechnol Pharm.

[11] Igarashi Y, Kuwamori Y, Takagi K, Ando T, Fudou R, Furumai T, Oki T (2000). Xanthoepocin, a new antibiotic from *Penicillium simplicissimum* IFO5762. J Antibiot (Tokyo).

[12] https://adisinsight.springer.com/drugs/800014881. Accessed 18 Aug 2021.

[13] Blank F, Buxtorf C, Chin O, Just G, Tudor JL (1969). Metabolites of pathogenic fungi. VIII. Floccosin and floccosic acid, two metabolites from *Epidermophyton floccosum*. Can J Chem.

[14] Brisse F, Just G, Blank F (1978). Metabolites of pathogenic fungi. IX. The crystal and molecular structure of floccosin, C30H26O14, and some of its degradation products. Acta Crystallogr Sect B.

[15] Kawai K, Nakamaru T, Nozawa Y (1982). The biological activity of floccosin from *Epidermophyton floccosum*. The effects on mitochondrial reactions. Maikotokishin (Tokyo).

[16] Mori H, Kawai K, Ohbayashi F, Kitamura J, Nozawa Y (1983). Genotoxicity of quinone pigments from pathogenic fungi. Mutat Res Lett.

[17] Mori H, Kawai K, Ohbayashi F, Kuniyasu T, Yamazaki M, Hamasaki T, Williams GM (1984). Genotoxicity of a variety of mycotoxins in the hepatocyte primary culture/DNA repair test using rat and mouse hepatocytes. Cancer Res.

[18] Song Y, Dou H, Wang P, Zhao S, Wang T, Gong W, Zhao J, Li E, Tan R, Hou Y (2014). A novel small-molecule compound diaporine A inhibits non-small cell lung cancer growth by regulating miR-99a/mTOR signaling. Cancer Biol Ther.

[19] Wu HC, Ge HM, le Zang Y, Bei YC, Niu ZY, Wei W, Feng XJ, Ding S, Ng SW, Shen PP, Tan RX (2014). Diaporine, a novel endophyte-derived regulator of macrophage differentiation. Org Biomol Chem.

[20] Feng X, Yu W, Zhou F, Chen J, Shen P (2016). A novel small molecule compound diaporine inhibits breast cancer cell proliferation via promoting ROS generation. Biomed Pharmacother.

[21] Vil V, Gloriozova TA, Poroikov VV, Terent'ev AO, Savidov N, Dembitsky VM (2019). Naturally occurring of alpha, beta-diepoxy-containing compounds: origin, structures, and biological activities. Appl Microbiol Biotechnol.

[22] Bode: Synthese, Biosynthese und absolute Konfiguration von Vioxanthin. Albert-Ludwigs-Universität Freiburg 2007.

[23] Fürtges L, Obermaier S, Thiele W, Foegen S, Müller M (2019). Diversity in fungal intermolecular phenol coupling of polyketides: regioselective laccase-based systems. ChemBioChem.

[24] Martínez-Aldino IY, Villaseca-Murillo M, Morales-Jiménez J, Rivera-Chávez J (2021). Absolute configuration and protein tyrosine phosphatase 1B inhibitory activity of xanthoepocin, a dimeric naphtopyrone from *Penicillium* sp. IQ-429. Bioorgan Chem.

[25] ICH IoWS: ICH M10 Guideline 2019. https://www.ema.europa.eu/en/documents/scientific-guideline/draft-ich-guideline-m10-bioanalytical-method-validation-step-2b_en.pdf. Accessed 15 Dec 2021.

[26] Siewert B, Vrabl P, Hammerle F, Bingger I, Stuppner H (2019). A convenient workflow to spot photosensitizers revealed photo-activity in basidiomycetes. RSC Adv.

[27] Tisch D, Schmoll M (2010). Light regulation of metabolic pathways in fungi. Appl Microbiol Biotechnol.

[28] Keller NP, Turner G, Bennett JW (2005). Fungal secondary metabolism—from biochemistry to genomics. Nat Rev Microbiol.

[29] Vrabl P, Schinagl CW, Artmann DJ, Heiss B, Burgstaller W (2019). Fungal growth in batch culture—what we could benefit if we start looking closer. Front Microbiol.

[30] Fiala J, Schöbel H, Vrabl P, Dietrich D, Hammerle F, Artmann DJ, Stärz R, Peintner U, Siewert B (2021). A new high-throughput-screening-assay for photoantimicrobials based on EUCAST revealed unknown photoantimicrobials in cortinariaceae. Front Microbiol.

[31] Vrabl P, Mutschlechner W, Burgstaller W (2008). Characteristics of glucose uptake by glucose- and NH4-limited grown Penicillium ochrochloron at low, medium and high glucose concentration. Fungal Genet Biol.

[32] Olearo F, Both A, Belmar Campos C, Hilgarth H, Klupp EM, Hansen JL, Maurer FP, Christner M, Aepfelbacher M, Rohde H (2021). Emergence of linezolid-resistance in vancomycin-resistant Enterococcus faecium ST117 associated with increased linezolid-consumption. Int J Med Microbiol.

[33] Siewert B, Stuppner H (2019). The photoactivity of natural products—An overlooked potential of phytomedicines?. Phytomedicine.

[34] Comini LR, Núñez Montoya SC, Sarmiento M, Cabrera JL, Argüello GA (2007). Characterizing some photophysical, photochemical and photobiological properties of photosensitizing anthraquinones. J Photochem Photobiol A.

[35] Gmoser R, Ferreira JA, Lennartsson PR, Taherzadeh MJ (2017). Filamentous ascomycetes fungi as a source of natural pigments. Fungal Biol Biotechnol.

[36] Egli T (2015). Microbial growth and physiology: a call for better craftsmanship. Front Microbiol.

[37] Daub ME, Chung K-R (2007). Cercosporin: A photoactivated toxin in plant diseas. Online APSnet Features.

[38] de Jonge R, Ebert MK, Huitt-Roehl CR, Pal P, Suttle JC, Spanner RE, Neubauer JD, Jurick WM, Stott KA, Secor GA (2018). Gene cluster conservation provides insight into cercosporin biosynthesis and extends production to the genus Colletotrichum. Proc Natl Acad Sci U S A.

[39] Kalra R, Conlan XA, Goel M (2020). Fungi as a potential source of pigments: harnessing filamentous fungi. Front Chem.

[40] Meruvu H, Dos Santos JC (2021). Colors of life: a review on fungal pigments. Crit Rev Biotechnol.

[41] Tacconelli E, Carrara E, Savoldi A, Harbarth S, Mendelson M, Monnet DL, Pulcini C, Kahlmeter G, Kluytmans J, Carmeli Y (2018). Discovery, research, and development of new antibiotics: the WHO priority list of antibiotic-resistant bacteria and tuberculosis. Lancet Infect Dis.

[42] Krull M, Klare I, Ross B, Trenschel R, Beelen DW, Todt D, Steinmann E, Buer J, Rath PM, Steinmann J (2016). Emergence of linezolid- and vancomycin-resistant *Enterococcus faecium* in a department for hematologic stem cell transplantation. Antimicrob Resist Infect Control.

[43] Klupp EM, Both A, Belmar Campos C, Buttner H, Konig C, Christopeit M, Christner M, Aepfelbacher M, Rohde H (2016). Tedizolid susceptibility in linezolid- and vancomycin-resistant *Enterococcus faecium* isolates. Eur J Clin Microbiol Infect Dis.

[44] Decousser JW, Woerther PL, Soussy CJ, Fines-Guyon M, Dowzicky MJ (2018). The tigecycline evaluation and surveillance trial; assessment of the activity of tigecycline and other selected antibiotics against gram-positive and gram-negative pathogens from France collected between 2004 and 2016. Antimicrob Resist Infect Control.

[45] EUCAST Clinical breakpoints—breakpoints and guidance. https://www.eucast.org/clinical_breakpoints/. Accessed 15 Dec 2021.

[46] Meijer MS, Bonnet S (2019). diastereoselective synthesis and two-step photocleavage of ruthenium polypyridyl complexes bearing a bis(thioether) ligand. Inorg Chem.

[47] Vrabl P, Fuchs V, Pichler B, Schinagl CW, Burgstaller W (2012). Organic acid excretion in *Penicillium ochrochloron* increases with ambient pH. Front Microbiol.

