# Supplementary material for: Xanthoepocin, a photolabile antibiotic of Penicillium ochrochloron CBS 123823 with high activity against multiresistant gram-positive bacteria
Source: Microb Cell Fact. 2022 Jan 4;21:1. doi: 10.1186/s12934-021-01718-9 (PMC8725544; doi:10.1186/s12934-021-01718-9)
Supplement: Supplementary file 1 — Additional file 1. Additional tables and and figures. [file 12934_2021_1718_MOESM1_ESM.docx]

# ADDITIONAL FILES 1 to the Manuscript

**Xanthoepocin, a photolabile antibiotic of *Penicillium ochrochloron* CBS 123.823 with high activity against multiresistant gram-positive bacteria**

Pamela Vrabl, Bianka Siewert, Jacqueline Winkler, Harald Schöbel, Christoph W. Schinagl, Ludwig Knabl, Dorothea Orth-Höller, Johannes Fiala, Michael Meijer, Sylvestre Bonnet, Wolfgang Burgstaller


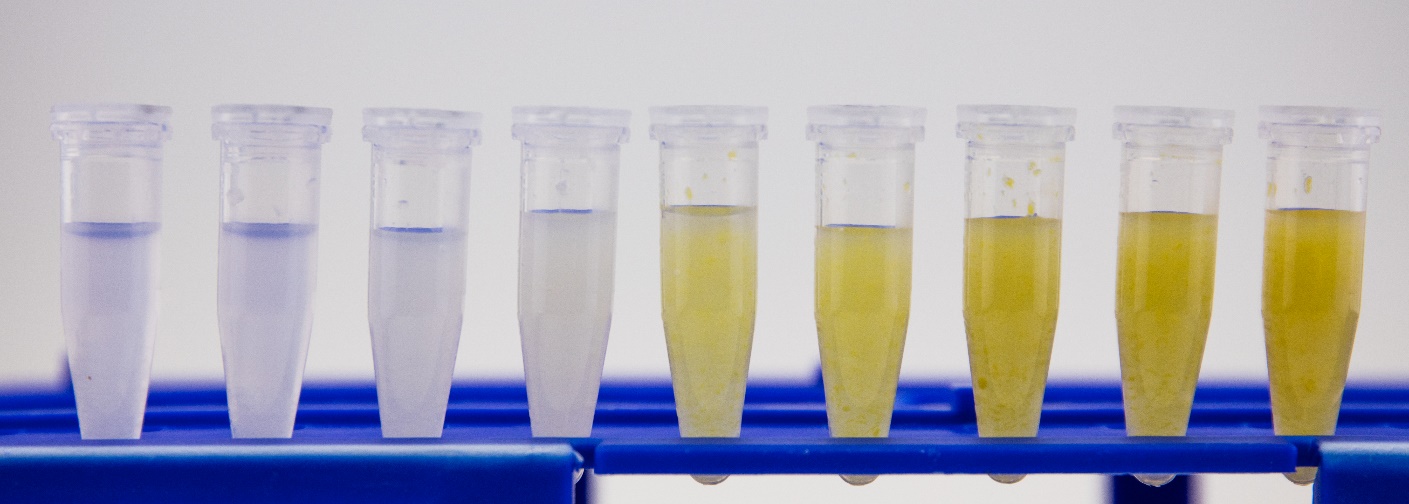


## Chemical characterization

### Calibration Curve Xanthoepocin


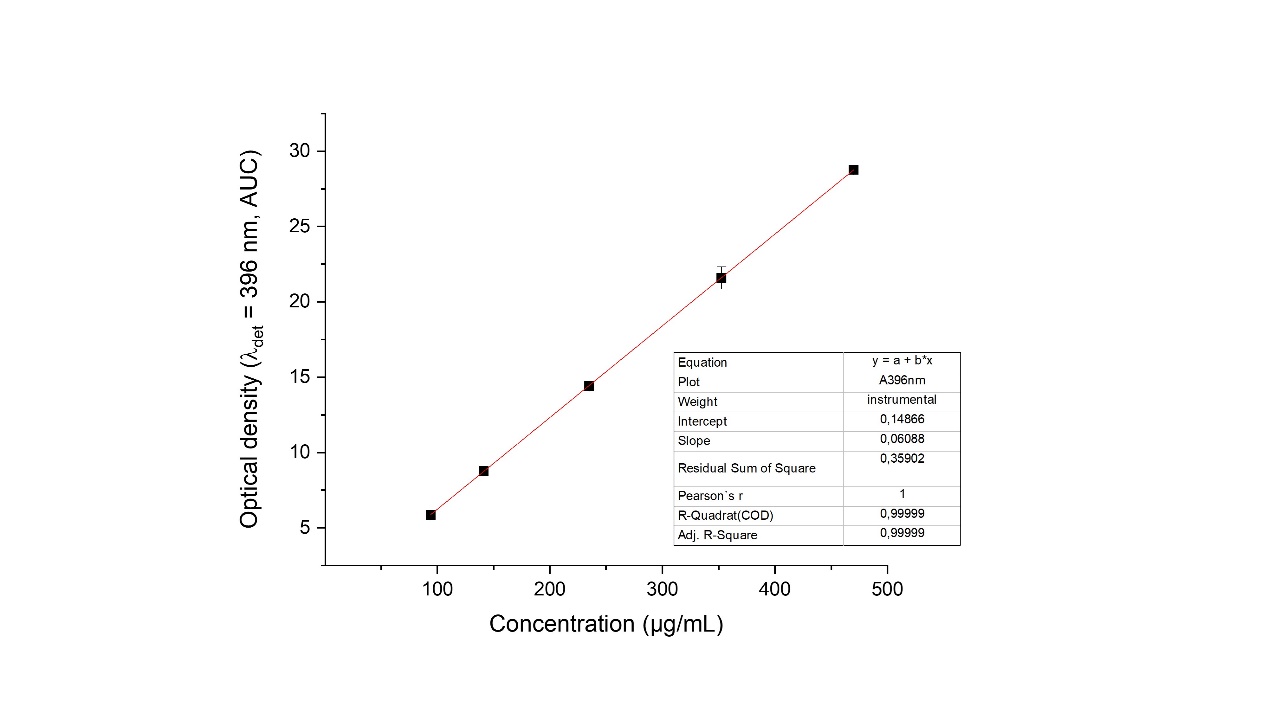


Additional file 1: Figure S1: Calibration curve xanthoepocin with embedded result of the linear regression.

### Chemical Characterization of Xanthoepocin (1)

Xanthoepocin was obtained as light-yellow solid. R_f_ (SiO_2_, CHCl_3_/MeOH 20:1, developed with EtOH/H^+^) = 0.46; λ_max_ (MeOH, ε in cm mol^-^ L^-^) = 399 (4795) nm; IR (cm^-1^) = 1704*m*, 1682*s*, 1646*s*, 1618*m*, 1558*w*, 1426*w*, 1384*w,* 1324*w*, 1263*m*, 1232*m*, 1200*w*, 1149*s*, 1092*m*, 1055*w*, 1011*m*, 986*w*, 920*w*, 820*w*, 794*w*, 739*w*, 723*w*, 502*w*. 486*w*; ^1^H-NMR (600 MHz, DMSO) = δ 7.53 (*s*, 1H, C*H* (5)), 6.78 (*d*, J = 1.2 Hz, 1H, C*H* (4)), 5.25 (*s*, 1H, C*H* (9)), 3.63 (s, 3H, OC*H*_3_), 2.28 (*d*, J = 1.0 Hz, 3H, C*H*_3_ (12)) ppm; ESI-MS (m/z, pos) = 607.1 ([M+H]^+^, 100%); ESI-MS (m/z, neg) = 605.0 ([M-H]^-^), 623.3 ([M-H+H_2_O]^-^).


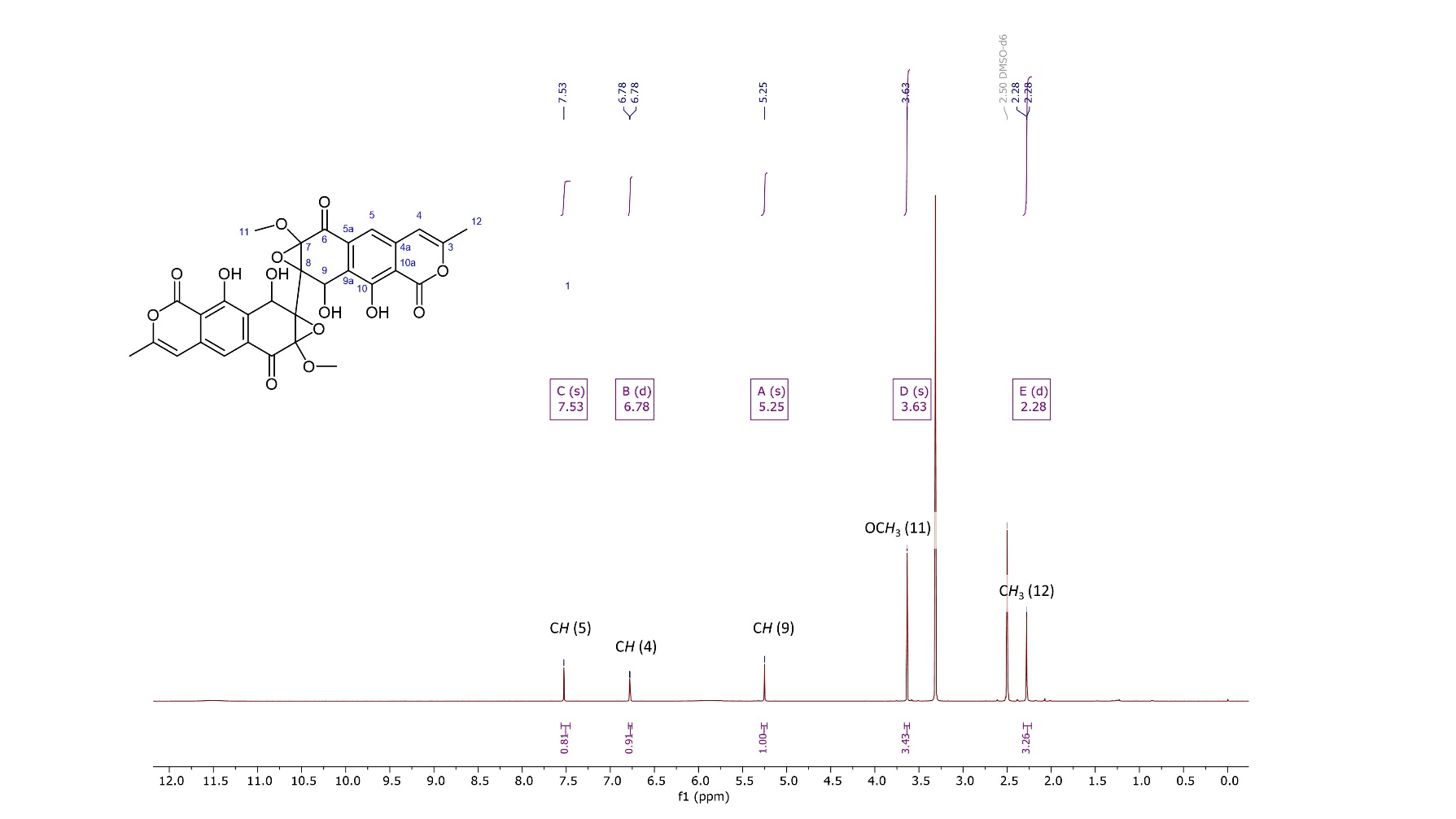


Additional file 1: Figure S2: H-NMR (600 MHz, d6-DMSO) of xanthoepocin with the assignment of the protons.


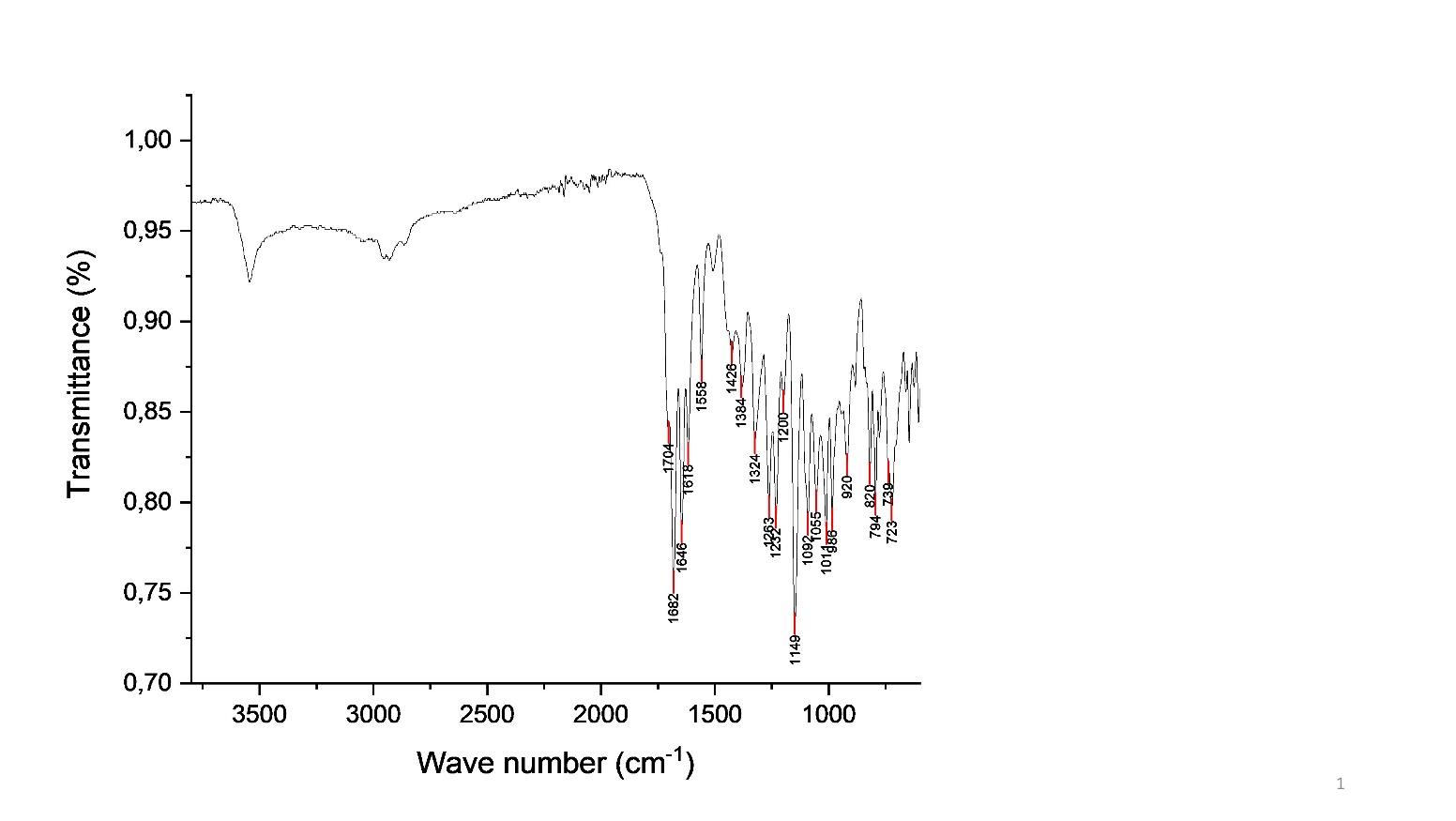


Additional file 1: Figure S3: FT-IR spectra of xanthoepocin.


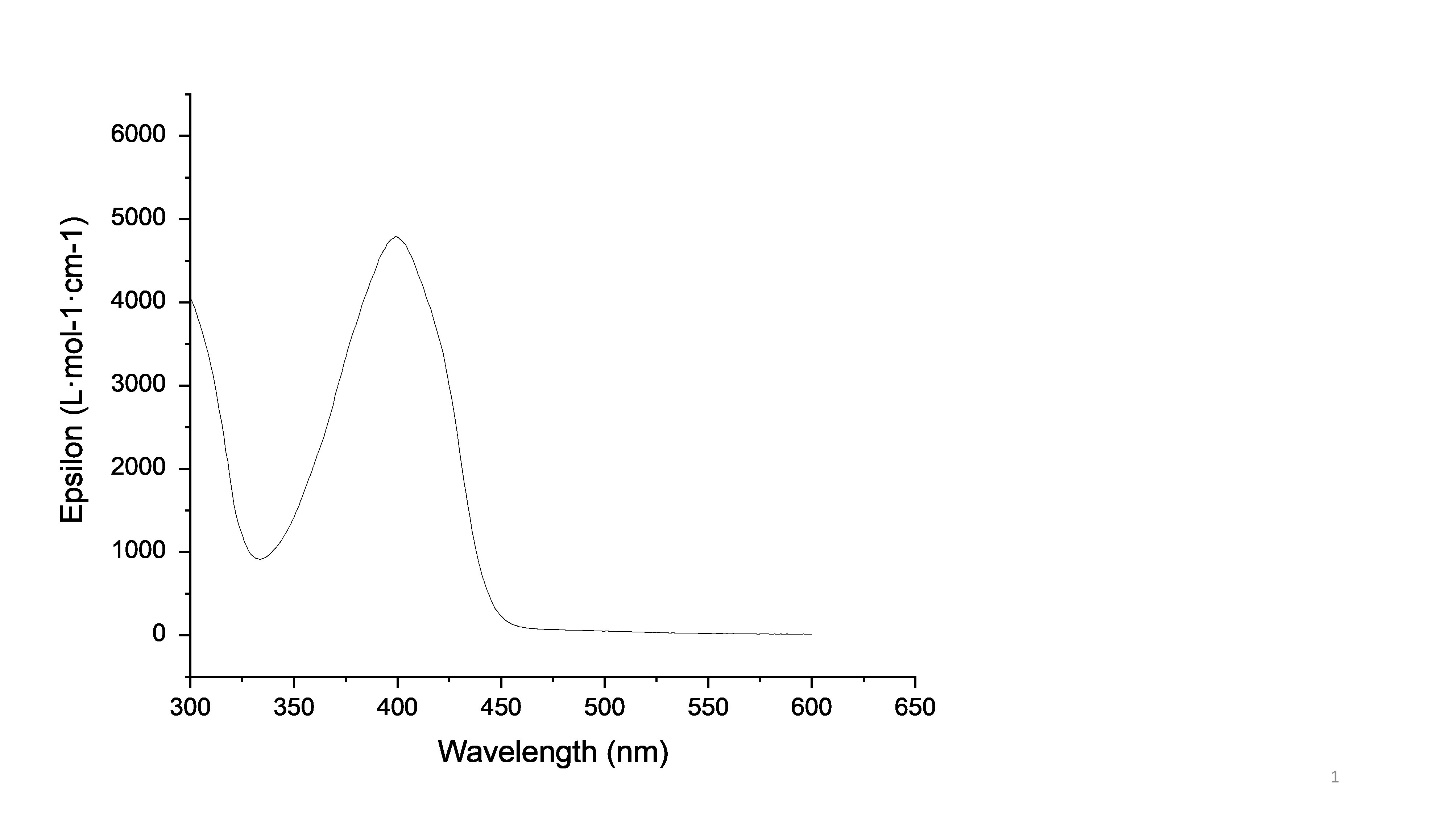


Additional file 1: Figure S4: Absorption spectra of xanthoepocin measured in methanol. Depicted is the molar attenuation coefficient versus the wavelength. Concentration range used: 8 µM - 400 µM.

### Aggregation behaviour

Additional file 1: Figure S5: Size distribution plot of the aggregate structures observed in Müller Hilton broth (red) and formed by xanthoepocin (green c = 500 µM, blue c = 5 µM) in the buffer.


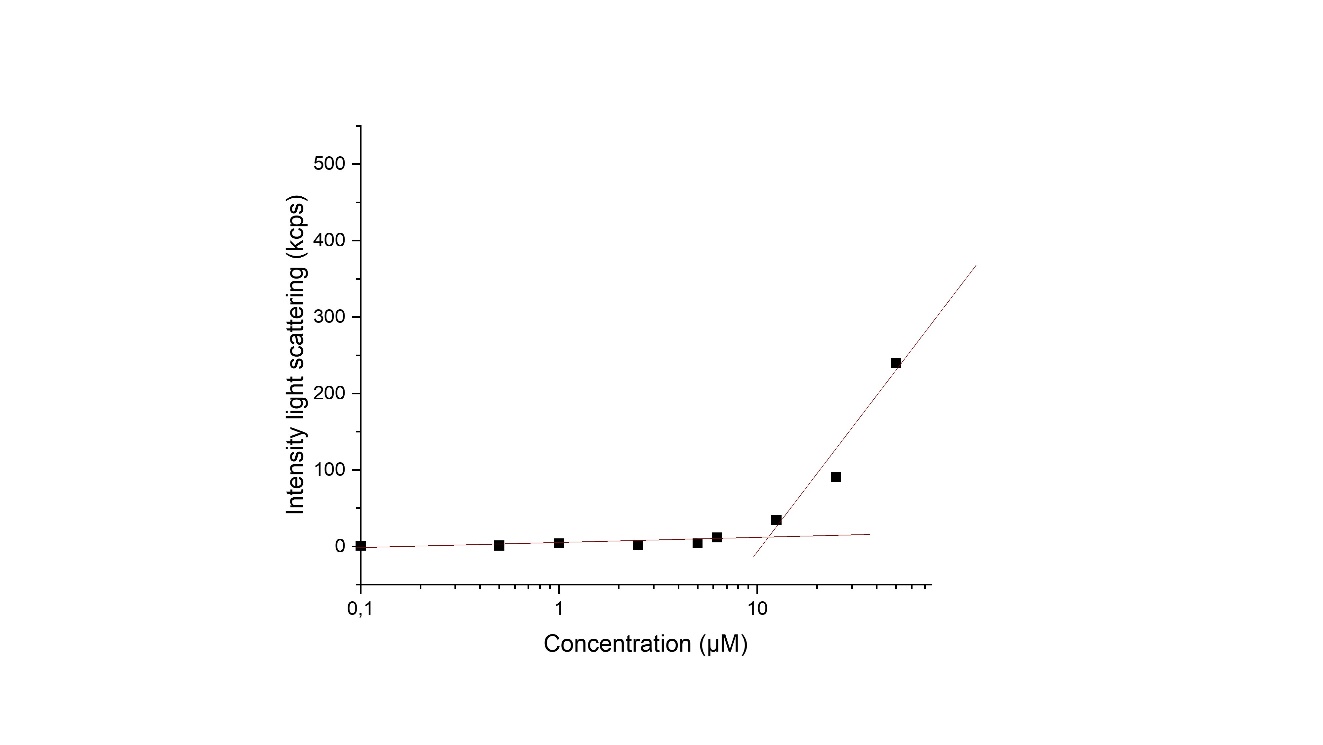


Additional file 1: Figure S6: Representative plot of the intensity of scattered light (in kilocounts per second) obtained by DLS for samples containing various concentrations of xanthoepocin in PBS. The intersection corresponds to the critical aggregate concentration (CAC).

### Photochemical Characterization


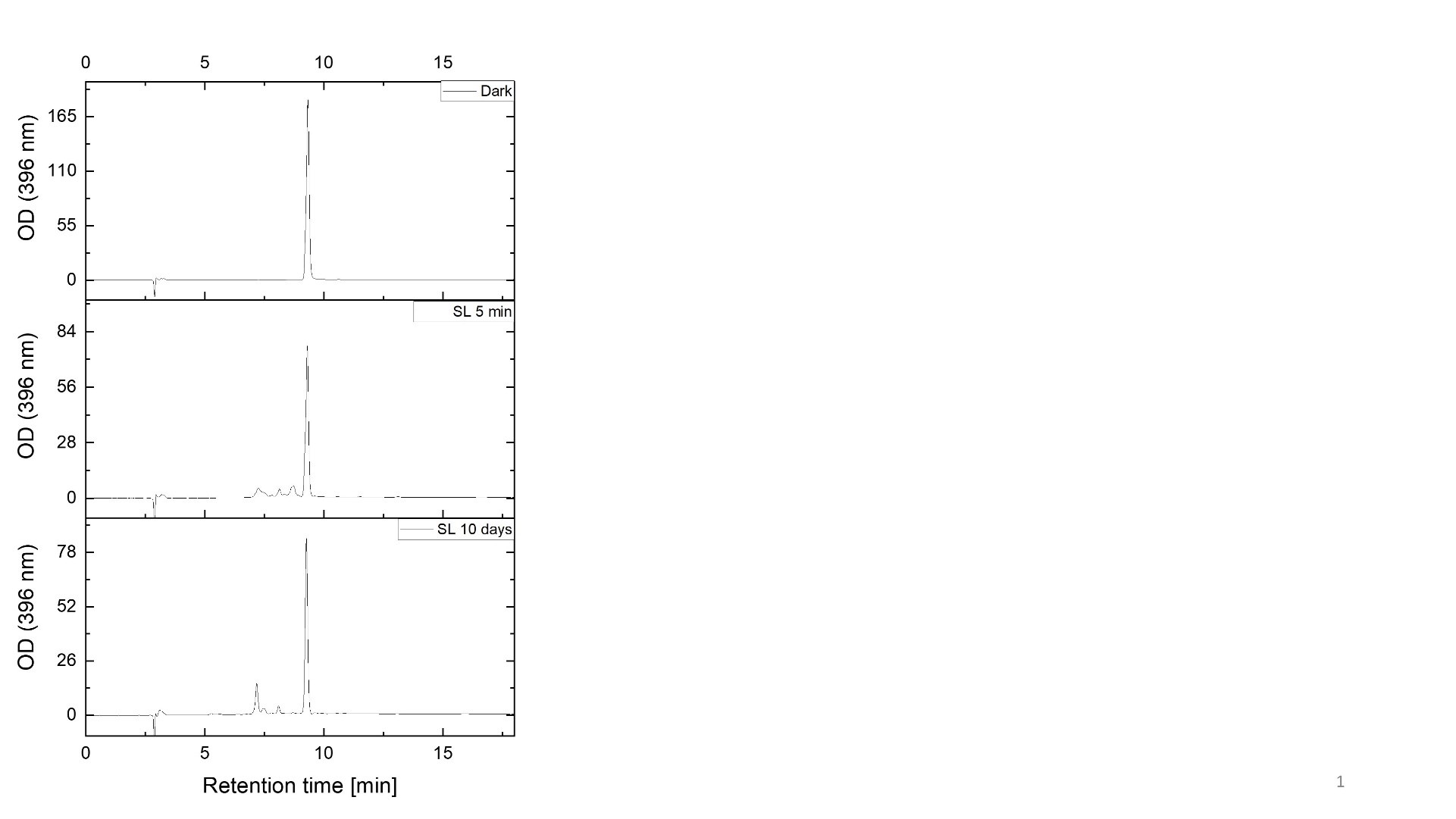


Additional file 1: Figure S7: HPLC-DAD investigation of sun-light irradiated xanthoepocin samples as compared to the pure compound in the dark (upper panel).


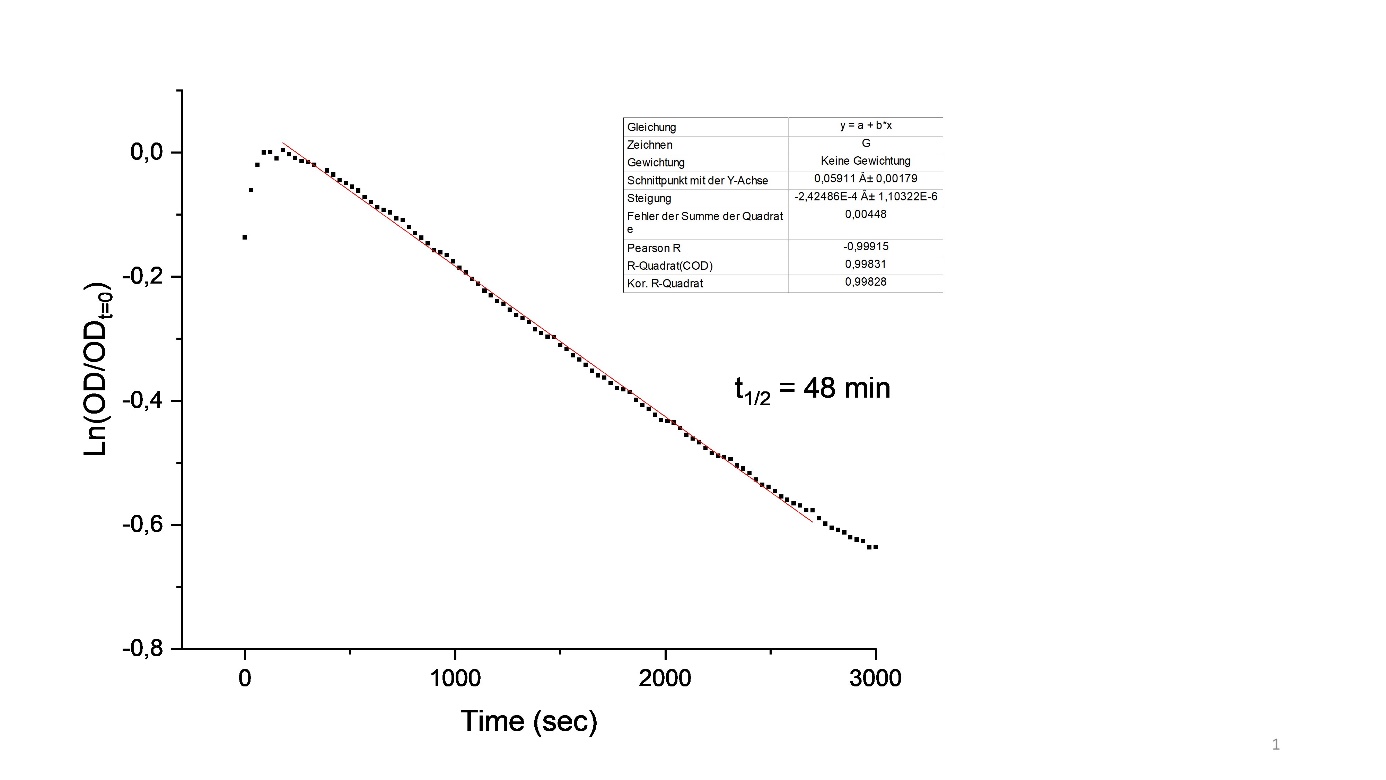


Additional file 1: Figure S8: Degradation studies of xanthoepocin under blue light irradiation.


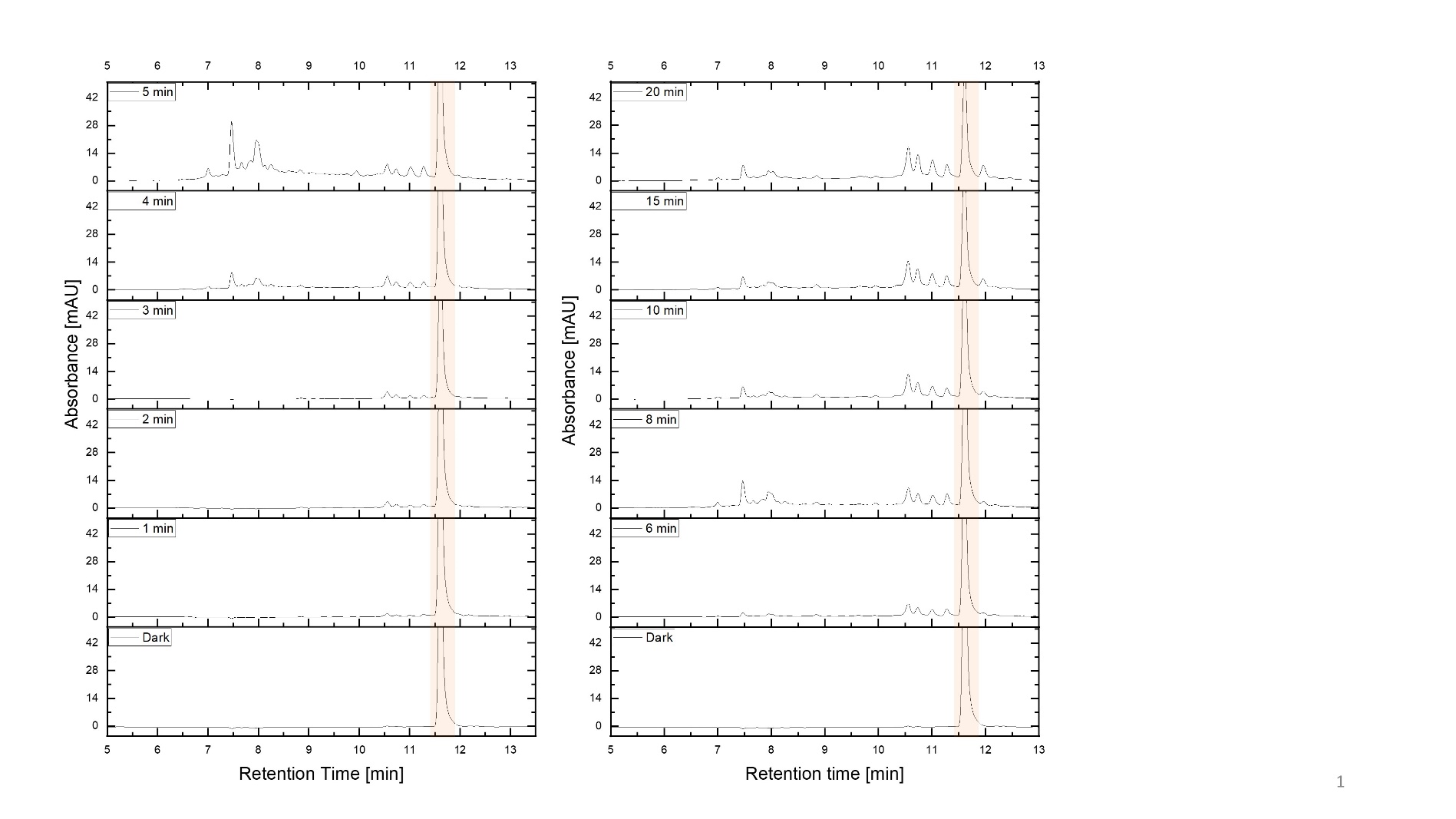


Additional file 1: Figure S9: Evolution of xanthoepocin’s photoproducts under blue light irradiation (λ = 450±50 nm). Xanthoepocin is orange highlighted.


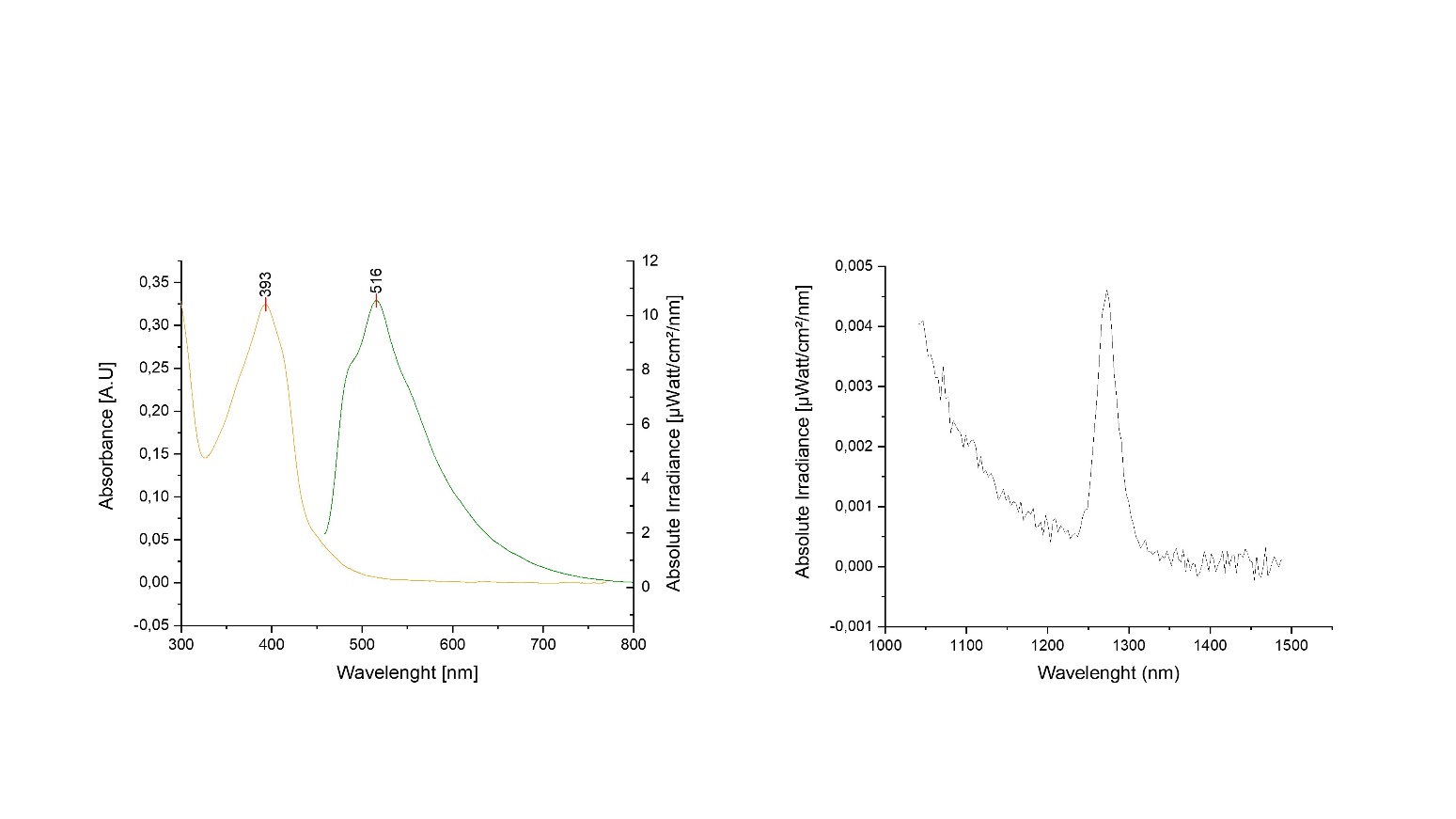


**A**

**B**

Additional file 1: Figure S10: A) Absorbance and emission spectra of xanthoepocin in deuterated methanol (λ_exc_ = 450 nm). B) Near infra-red emission of ^1^O_2_ measured after excitation of xanthoepocin (λ_exc_ = 450 nm, d_4_-MeOH).


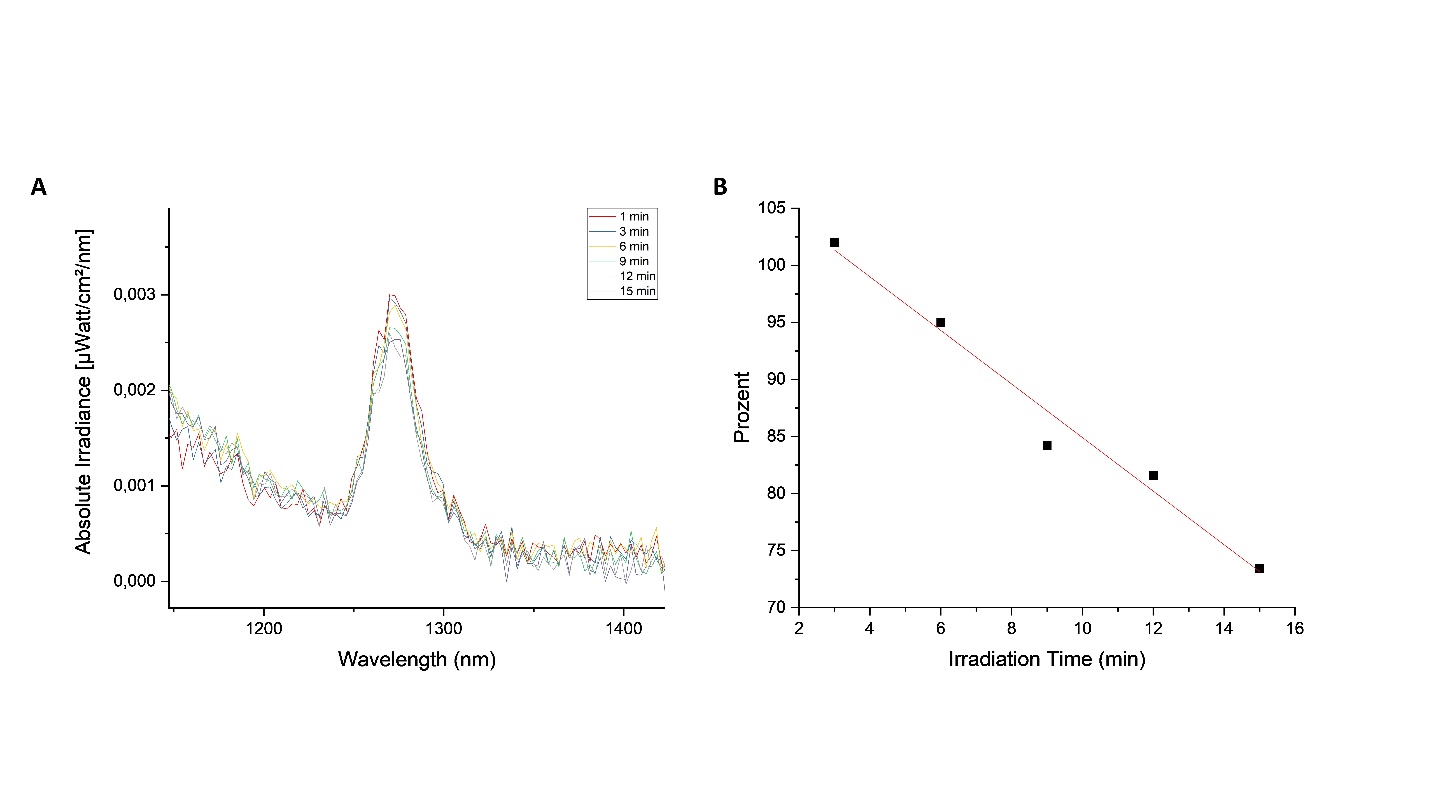


Additional file 1: Figure S11: Quantification of ^1^O_2_ production over time. A) Near read emission of ^1^O_2_ measured after repeated excitation of xanthoepocin (λ_exc_ = 450 nm, d_4_-MeOH), B) correlation of ^1^O_2_ production by xanthoepocin in percent vs time.

## Petri Dish Experiments

Additional file 1: Table S1: Medium composition for the petri-dish experiments (modified after Vrabl et al. 2019)

| Compound | C-lim | P-lim | N-lim |
| --- | --- | --- | --- |
|  | mM | mM | mM |
|  |  |  |  |
| D(+)Glucose · H_2_O | 20 | 400 | 400 |
| (NH_4_)_2_SO_4_ | 6.25 | 6.25 | 5 |
| NH_4_Cl | 12.50 | 12.50 | 10 |
| KH_2_PO_4_ | 5.80 | 0.50 | 5.80 |
| MgSO_4_ · 7H_2_O | 1.60 | 1.60 | 1.6 |
| KCl | - | 5.30 | - |
| Agar-Agar | 18 | 18 | 18 |
| 10 ml trace element solution (double concentrated) | | | |

The pH was adjusted with 1 M NaOH to pH 7.


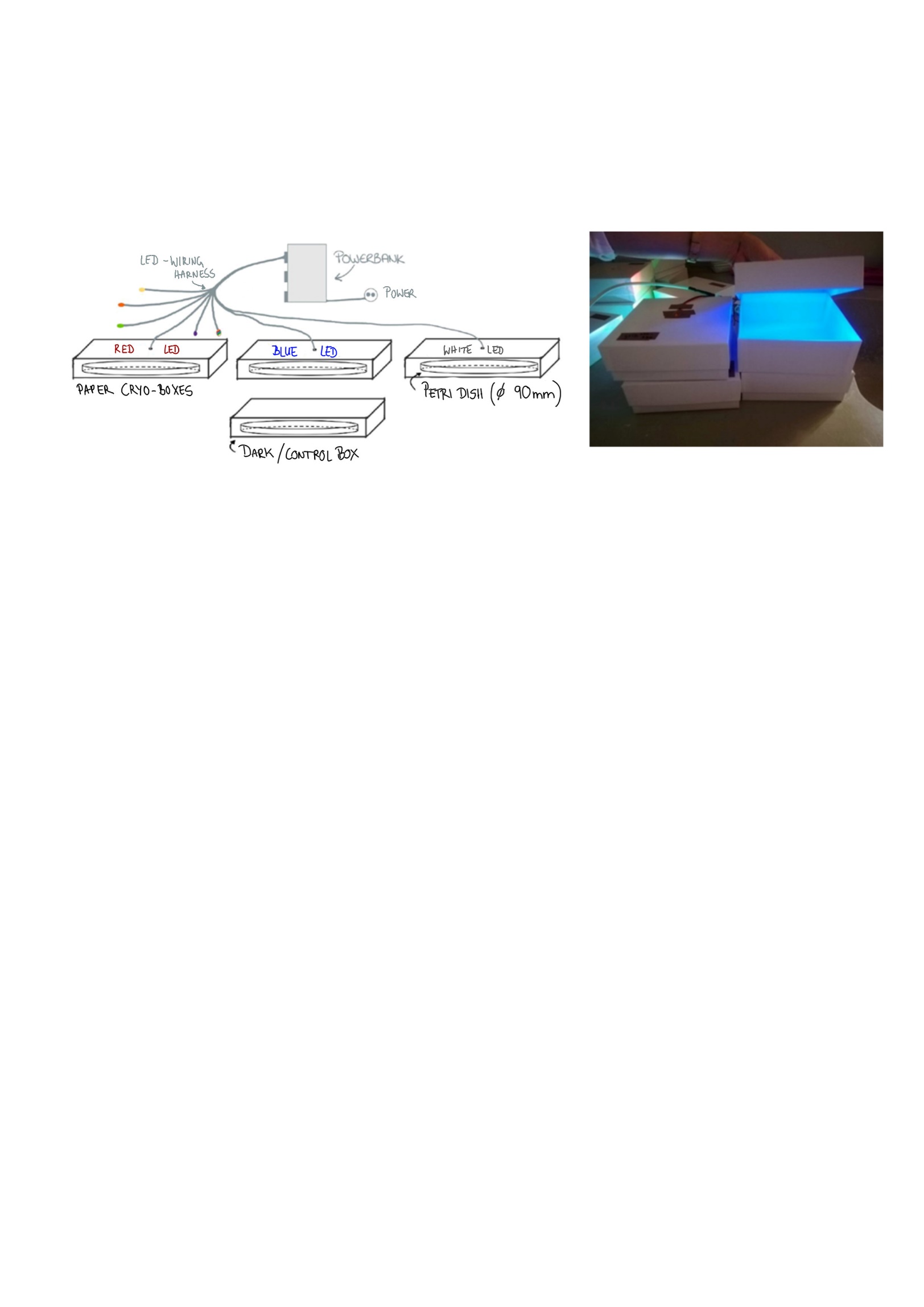


**Additional file 1: Figure S12:** Experimental setup for the Petri dish experiments comprising light-impermeable carton boxes, each with one light-emitting diode (LED). *Left*: Overall schematics (with kind permission from Kim Oun). *Right*: Photo of the experimental setting.

Additional file 1: Table S2: Central wavelengths, spectral width (full width at half of the intensity maximum, FWHM) and intensity range applied to the petri dish experiments.

| Color | $\lambda_{peak}$ | $\Delta\lambda$  (FWHM) | min intensity | max intensity |
| --- | --- | --- | --- | --- |
|  | nm | nm | mw cm^-2^ | mw cm^-2^ |
| violet | 394 | 10 | 0.80 | 1.20 |
| blue | 464 | 18 | 0.60 | 1.20 |
| green | 519 | 28 | 0.30 | 0.50 |
| yellow | 593 | 11 | 0.02 | 0.05 |
| orange | 608 | 14 | 0.10 | 0.20 |
| red | 631 | 17 | 0.00 | 0.30 |
|  |  |  |  |  |


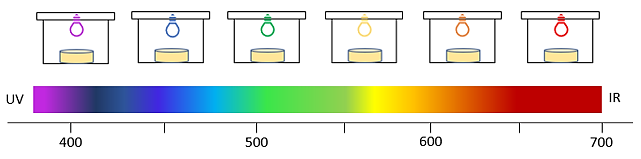

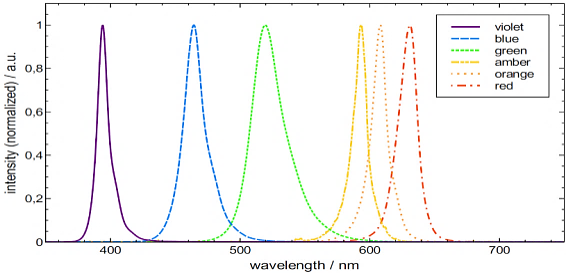


**B**

**A**

**Additional file 1: Figure S13:** Experimental setup for the explorative screening on petri dishes. **(A)** One LED per irradiation condition was placed in a light-impermeable carton box (see also Additional file 1: Figure S12). **(B)** Spectral distribution of the different irradiation scenarios (further information see also Additional file 1: Table S2). For better comparison, intensities were normalized.


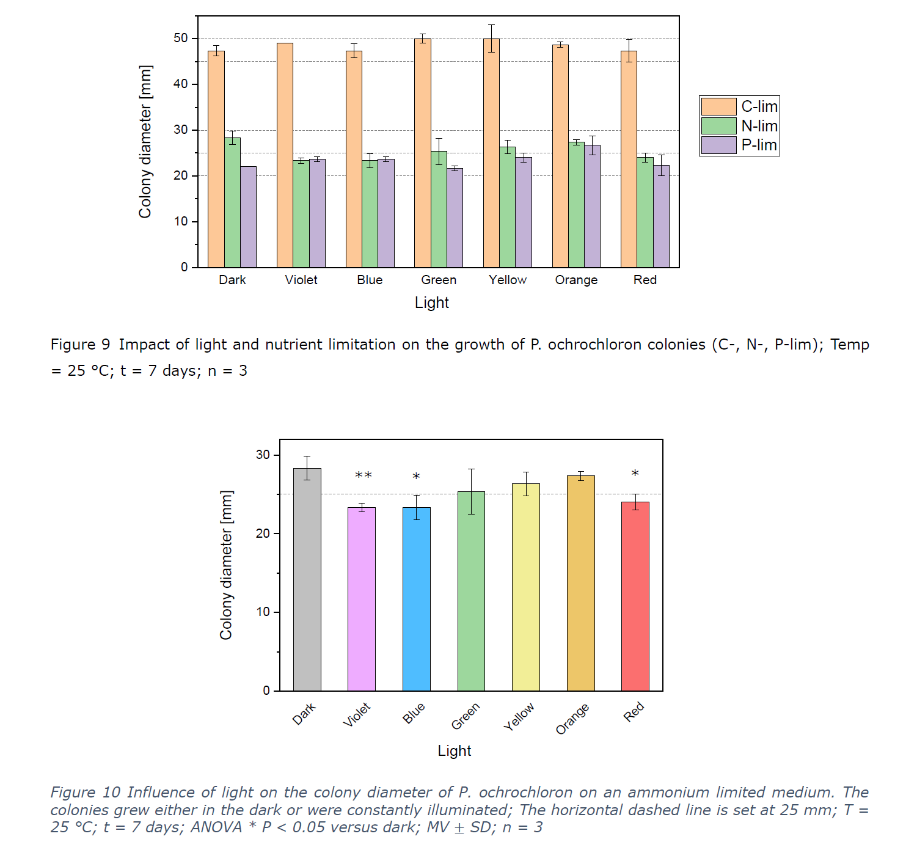


Additional file 1: Figure S14: Colony diameters of *P. ochrochloron* CBS 123823 cultures grown on carbon-, ammonium- or phosphate-limited conditions for 7 days at 25 °C in dependence of the irradiation conditions (n = 3).


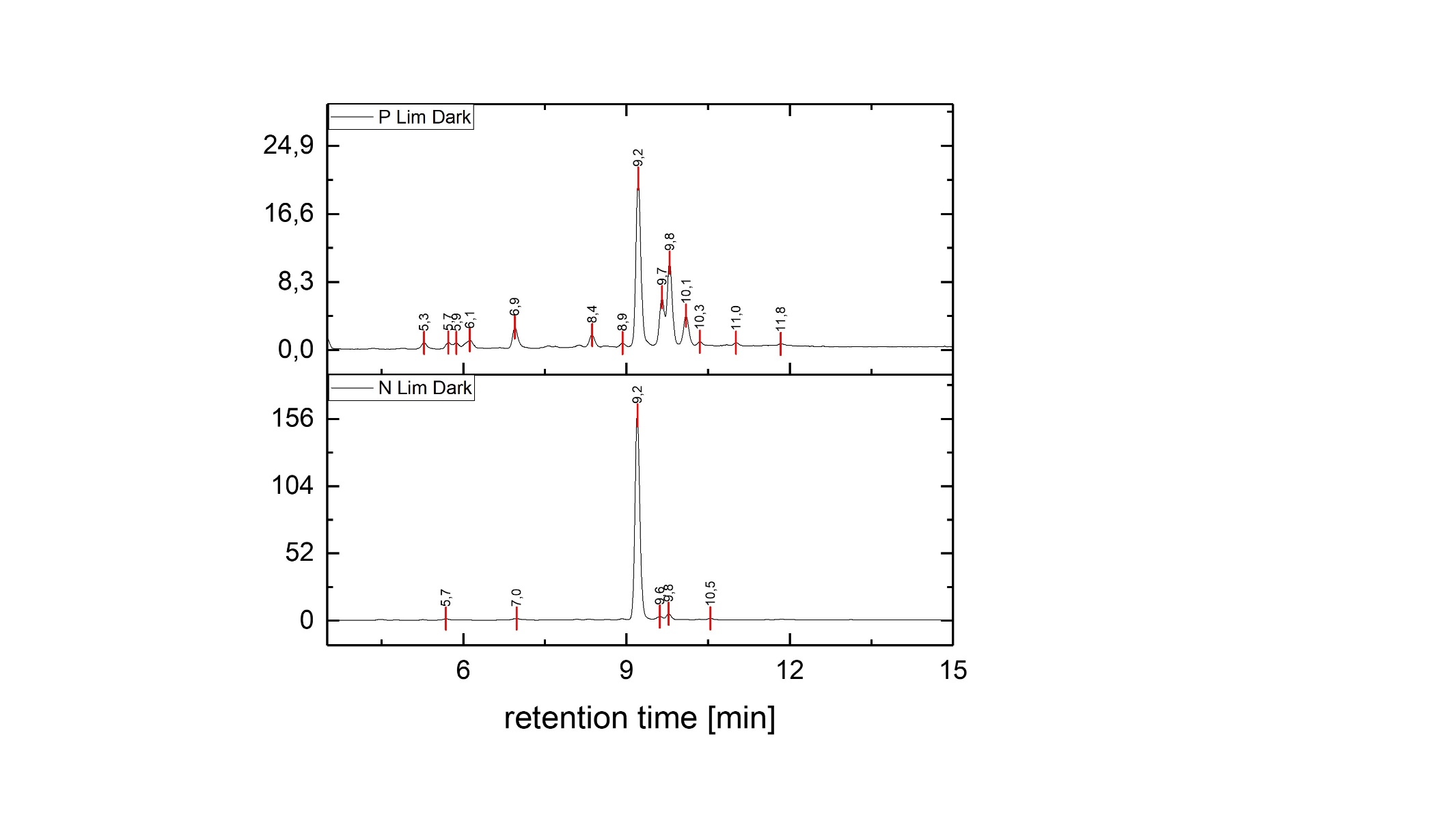


Additional file 1: Figure S15: Metabolite profile of (upper panel) phosphate limited and (lower panel) ammonium limited cultures grown in darkness. The retention time of xanthoepocin is 9.2 min. Number of visible peaks: P-lim 14 vs N-lim 6.

## BIOREACTOR BATCH experiments

**Additional file 1: Table S3**: Relevant illumination properties of the used light sources for the bioreactor batch experiments

|  | | | | | | |
| --- | --- | --- | --- | --- | --- | --- |
| Light source | Color | $\lambda_{peak}$ | $\Delta\lambda$  (FWHM) | Correlated color Temperature | Intensity | Photon flux  density |
|  |  | nm | nm | K | W m^-2^ | µmol s^-1^ m^-2^ |
| Philips TLD 18W/33-640 | cool white | - | - | 4100 | 0.3 | 2 |
| Osram Oslon SSL 150 | deep blue | 451 | 20 | - | 90 | 340 |
| Osram Oslon SSL 150 | hyper red | 660 | 25 | - | 60 | 330 |

**A**


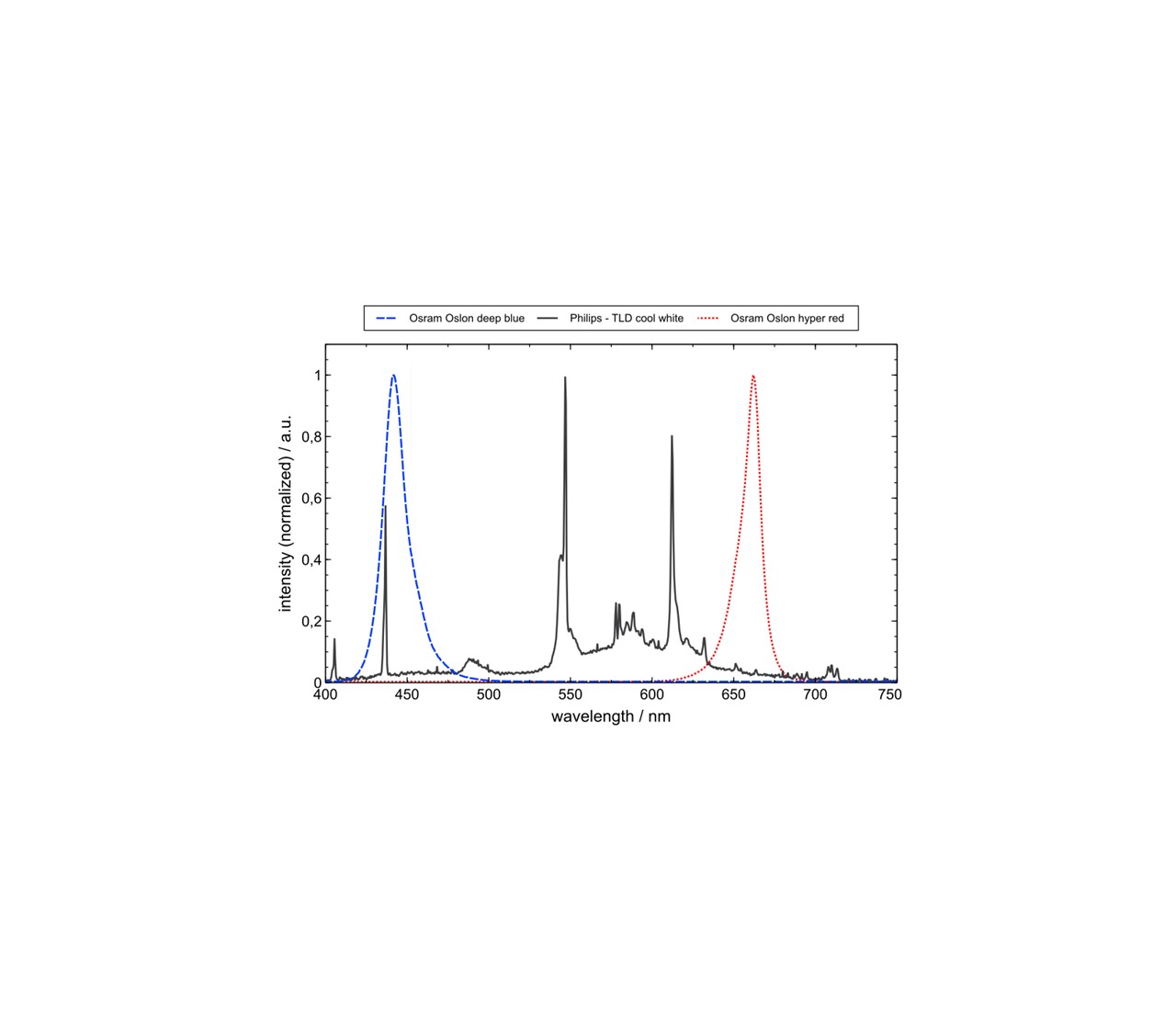


**B**


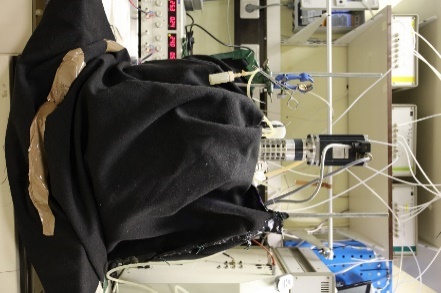

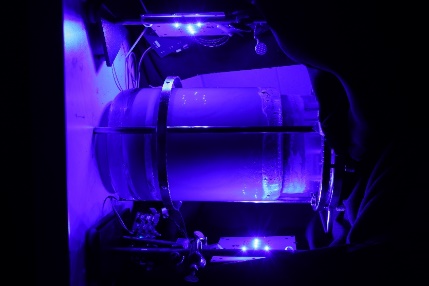

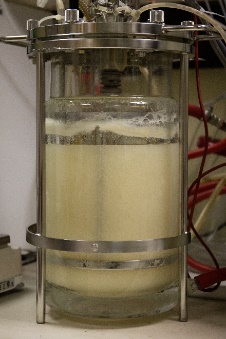

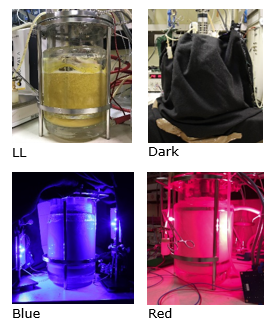


Additional file 1: Figure S16: Experimental setup for the bioreactor batch experiments. (A) From left to right: uncovered bioreactor; covered bioreactor illuminated with either red or blue light; covered bioreactor. (B) Spectral distribution of the different irradiation scenarios for cultivation experiments in bioreactor batch experiments. Blue light: Osram Oslon SSL 150 deep blue, λpeak= 451 nm. White light: fluorescent tube, Philips TLD, CCT 4100 K. Red light: Osram Oslon SSL 150 hyper red, λpeak= 660 nm. For better comparison, intensities were normalized.

Additional file 1: Figure S17: Biomass evolution and residual nutrient concentrations of an ammonium limited culture of *P. ochrochloron* CBS 123 823 grown in darkness. The dotted line marks the ammonium exhaustion in the medium. Data are means of three samples per sampling time point. A typical growth curve is shown. Symbols: ■ ammonium (NH_4_), △ dry matter (DM), ▼ glucose (Glc), and ⭘ phosphate (PO_4_).

Additional file 1: Figure S18: Biomass evolution and residual nutrient concentrations of an ammonium limited culture of *P. ochrochloron* CBS 123 823 grown under continuous red light irradiation. The dotted line marks the ammonium exhaustion in the medium. Data are means of three samples per sampling time point. A typical growth curve is shown. Symbols: ■ ammonium (NH_4_), △ dry matter (DM), ▼ glucose (Glc), and ⭘ phosphate (PO_4_).

Additional file 1: Figure S19: Biomass evolution and residual nutrient concentrations of an ammonium limited culture of *P. ochrochloron* CBS 123 823 grown under continuous ordinary laboratory light. The dotted line marks the ammonium exhaustion in the medium. Data are means of three samples per sampling time point. A typical growth curve is shown. Symbols: ■ ammonium (NH_4_), △ dry matter (DM), ▼ glucose (Glc), and ⭘ phosphate (PO_4_).

Additional file 1: Figure S20: Biomass evolution and residual nutrient concentrations of an ammonium limited culture of *P. ochrochloron* CBS 123 823 grown under continuous blue light irradiation. The dotted line marks the ammonium exhaustion in the medium. Data are means of three samples per sampling time point. A typical growth curve is shown. Symbols: ■ ammonium (NH_4_), △ dry matter (DM), ▼ glucose (Glc), and ⭘ phosphate (PO_4_).

## Photoantimicrobial Testing

**
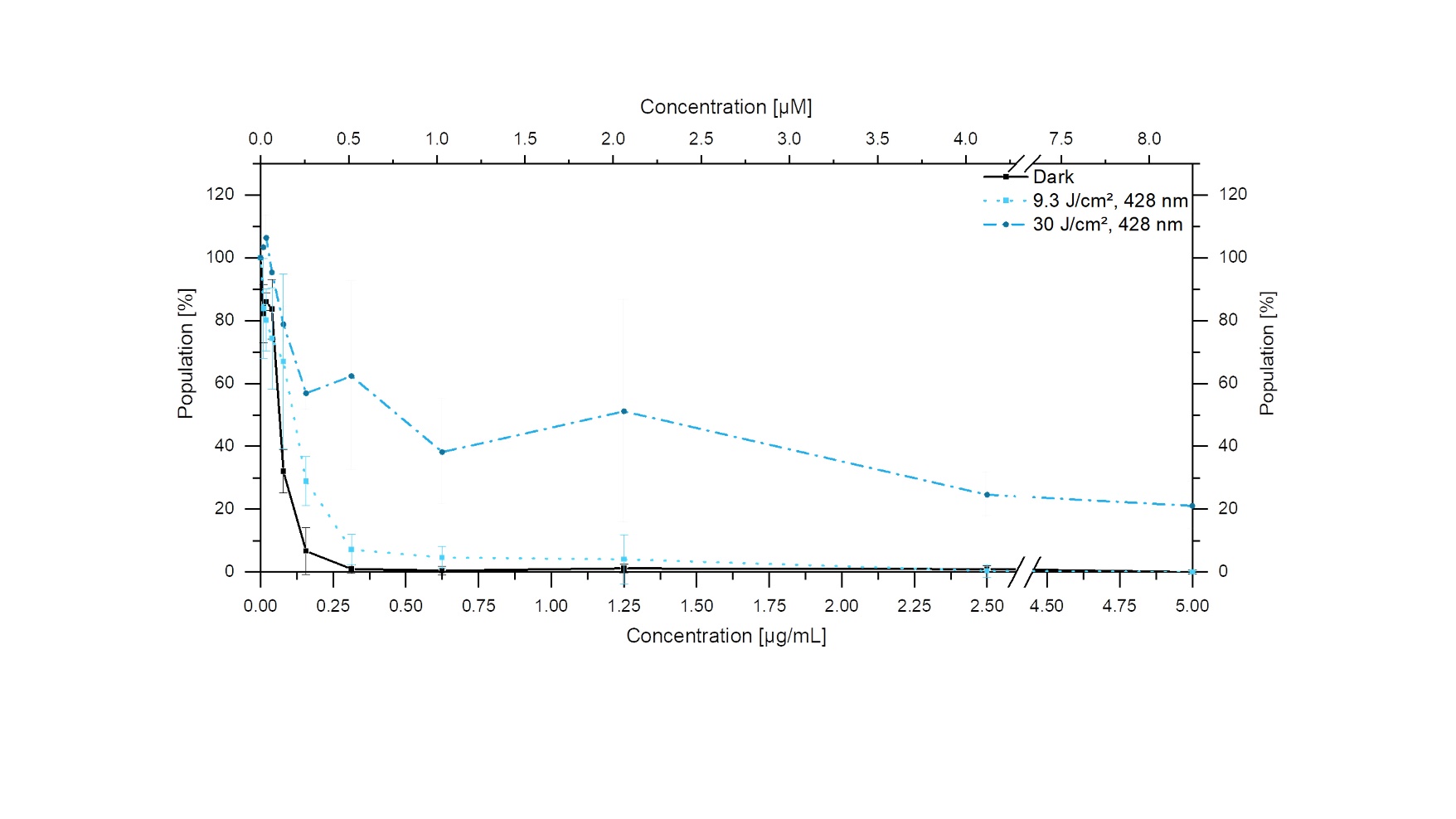
**

**Additional file 1: Figure S21:** Dose-response curve of xanthoepocin against a population of *S. aureus* (DSM1104) in the dark (black plot), under irradiation with a weak dose of blue light (light blue dots, λ = 428 nm, H = 9.3 J cm^-2^), or an intense dose of blue light (dark blue plot, λ = 428 nm, H = 30 J cm^-2^).
